# Molecular assessment of recombination processing across genetically diverse mouse strains reveals sexually dimorphic determinants of crossover distribution beyond chromosome length

**DOI:** 10.1093/molbev/msag130

**Published:** 2026-06-02

**Authors:** Tegan S Horan, Anna Wood, Stephanie Tanis, Carme Peirau-Gabarrell, Paula E Cohen

**Affiliations:** Department of Biomedical Sciences, Cornell University, Ithaca, NY 14853, USA; Cornell Reproductive Sciences Center, Cornell University, Ithaca, NY 14853, USA; Department of Biomedical Sciences, Cornell University, Ithaca, NY 14853, USA; Cornell Reproductive Sciences Center, Cornell University, Ithaca, NY 14853, USA; Department of Biomedical Sciences, Cornell University, Ithaca, NY 14853, USA; Cornell Reproductive Sciences Center, Cornell University, Ithaca, NY 14853, USA; Cornell Reproductive Sciences Center, Cornell University, Ithaca, NY 14853, USA; Department of Biomedical Sciences, Cornell University, Ithaca, NY 14853, USA; Cornell Reproductive Sciences Center, Cornell University, Ithaca, NY 14853, USA

**Keywords:** crossover, meiotic recombination, crossover interference, sexual dimorphism

## Abstract

Meiotic recombination generates crossovers (COs), reciprocal exchanges between homologous chromosomes critical for accurate chromosome segregation. Inappropriate CO frequency and distribution drive aneuploidy in human oocytes, with error rates up to 10-fold higher than in sperm despite females exhibiting higher CO frequencies. COs form in the context of the proteinaceous synaptonemal complex (SC) that tethers homologs during prophase I. SC length strongly correlates with CO number, and sexual dimorphism in recombination has long been attributed to longer SCs in females. However, this model is challenged by wild-derived PWD mice in which males consistently generate more COs despite having shorter SCs. Here, we exploit natural genetic variation among inbred mouse strains to dissect the structural and regulatory basis of sexually dimorphic CO regulation. Using cytological markers of SC assembly (SYCP3), recombination progression (RAD51, MSH4), class I CO designation (HEI10, MLH1/MLH3), and chiasmata, we show that SC length is not the sole predictor of CO number. PWD males exhibit stronger CO interference and higher CO number than females, despite reduced SC length. Notably, females show reduced efficiency in designating recombination intermediates to become COs, whereas PWD males display exceptional proficiency. Unexpectedly, although class II COs are rare, they play a disproportionate role in ensuring that every chromosome pair receives at least one CO, thereby safeguarding against aneuploidy. Together, these findings challenge the prevailing view that SC length is the primary determinant of sexually dimorphic CO rates and instead highlight sex-specific regulation of CO designation and pathway usage as key drivers of recombination outcomes.

## Introduction

Germ cells have a formidable task during meiosis: They must halve their chromosome number through two cell divisions without intervening DNA replication to ensure each resulting gamete (sperm or egg) inherits exactly one copy of each chromosome. To accomplish this, the two parental chromosomes (homologs) must recognize each other and remain paired throughout prophase I via two interconnected processes. First, a protein structure, the synaptonemal complex (SC), forms between and tethers homologs together. Second, crossing over creates reciprocal DNA exchanges (crossovers, COs) that physically link homologs. These COs serve multiple functions: They maintain pairing through metaphase I, facilitate proper orientation and spindle attachment, and ensure optimal tension forms across the centromeres/kinetochores for homologs to be pulled to opposite poles (Jang et al. 1995; Koehler et al. 1996; Smith and Nambiar 2020; Evatt et al. 2024). Crossing over is tightly regulated to ensure each homologous pair receives at minimum one obligate CO (assurance), without which chromosomes segregate stochastically and frequently produce chromosomally atypical (aneuploid) gametes (Gray and Cohen 2016; Pazhayam et al. 2021). In mammals, COs are equally critical during spermatogenesis and oogenesis, and both processes utilize the same core DNA repair machinery to produce COs from an abundant pool of precursor events (Morelli and Cohen 2005). However, CO regulation shows considerable sexual dimorphism and produces male-female differences in both the number of COs (heterochiasmy) and their distribution along the chromosome (Cahoon and Libuda 2019; Sardell and Kirkpatrick 2020). Importantly in humans, these sex differences contribute to a high incidence of aneuploidy (roughly 10% of conceptuses), most originating from errors in maternal meiosis; as many as 20% to 80% of eggs (depending on age) contrasted with 2.5% to 7% of sperm have segregation errors (Nagaoka et al. 2012; Gruhn et al. 2019; Wartosch et al. 2021; Gruhn and Hoffmann 2022). Achiasmy (homologous chromosomes that fail to make the obligate CO) is the best-characterized source of aneuploidy in humans (Hassold and Hunt 2001; Nagaoka et al. 2012). Recent studies have shown that achiasmy is both common in human oocytes and its incidence is higher in oocytes with reduced global CO rates (Hassold et al. 2021). Importantly, growing evidence indicates that sub-optimal CO positioning (e.g. spaced too close together or too near the centromere or telomere) also strongly associates with maternal segregation errors (Nagaoka et al. 2012; Gruhn et al. 2019; Hassold and Hunt 2021).

Much of our understanding of the molecular regulation of CO processing comes from biochemical studies of *S. cerevisiae*, with confirmation of shared regulatory events in many meiotic species (Hunter 2015; Zickler and Kleckner 2015; Gray and Cohen 2016). In all cases, SPO11 and its accessory proteins induce programmed double-strand breaks (DSBs) throughout the genome at the onset of meiosis (Keeney et al. 1997; Garcia et al. 2011; Johnson et al. 2021). Of more than 200 breaks made in mice and humans, only ∼10% are resolved as COs (Zickler and Kleckner 2015; Gray and Cohen 2016). The remaining DSBs become non-crossovers (NCOs) via synthesis-dependent strand annealing (SDSA) or the helicase-mediated dissolution of repair intermediates (McMahill et al. 2007; Huselid et al. 2020; Sanchez et al. 2021; Hodson et al. 2022). Of the DSBs that resolve as COs, nearly all (90% to 95%) form via the class I pathway mediated by DNA mismatch repair (MMR) proteins: During CO licensing, MutSγ (MSH4-MSH5) binds and stabilizes repair intermediates, and a subset of these undergo designation and are resolved by MutLγ (MLH1-MLH3) as COs (Edelmann et al. 1999; Kneitz et al. 2000; Lipkin et al. 2002; Kolas et al. 2005; Manhart et al. 2017; Milano et al. 2019; Sanchez et al. 2021; Roy et al. 2025). Most of the remaining 5% to 10% of COs are the result of the minor class II pathway and mediated by the structure-selective nuclease, MUS81-EME1 (Holloway et al. 2008; Oh et al. 2008; Gray and Cohen 2016).

Both the number and the distribution of COs show striking sex differences: In most mammals, females have more COs than males, and males have more distally positioned COs than females (Morelli and Cohen 2005; Gruhn et al. 2013; de Boer et al. 2015; Sardell and Kirkpatrick 2020). Meiotic recombination occurs specifically within the context of the chromosome axis, comprised of an array of chromatin loops anchored to a linear proteinaceous scaffold comprised (Zickler and Kleckner 2015; Ishiguro 2019; Huang and Roig 2023). The number and spacing of class I COs along the axis are restricted by interference, a process that leads to the inhibition of two adjacent class I COs from forming within a close physical distance that is mediated in part by the SC (Sym and Roeder 1994; Otto and Payseur 2019; von Diezmann and Rog 2021; Durand et al. 2022; Fozard et al. 2022; Ernst et al. 2024). It is perhaps not surprising, therefore, that SC length also exhibits sexual dimorphism. Most female mammals have longer SCs than males, and a strong covariation between CO frequency and SC length is well established (Kleckner et al. 2003; Tease and Hultén 2004; Lynn et al. 2005; Gruhn et al. 2013; Ruiz-Herrera et al. 2017; Wang et al. 2019). SC length is also inversely correlated with chromatin loop size, and modeling of human meiosis has suggested modulation of loop size may regulate CO rate (Wang et al. 2017, 2021). Mouse studies have supported this notion as short chromosomes have disproportionately long axis lengths and smaller loops that may be necessary for sufficient numbers of induced DSBs to ensure the obligate CO is made (Kauppi et al. 2011; Murakami et al. 2020). In humans and mice, sex differences in interference distance tend to be larger when measured in genomic distance (megabases of DNA); however, inter-CO spacing is largely comparable at the level of micron length along the SC, leading to the long-held understanding that sex differences in CO frequency are attributable to sex differences in SC length (Lynn et al. 2002; Tease and Hultén 2004; Petkov et al. 2007; Wang et al. 2017; Ernst et al. 2024).

Most of what is known about the regulation of meiotic recombination in mammals comes from studies of mice, especially the C57BL/6J strain. Further, due to the difficulty inherent to female prophase I studies—these events arising during embryogenesis in females—most mammalian data come from investigations of spermatogenesis. Sex and strain differences in CO rates have previously been defined, but few studies have characterized strain differences in DSB formation or CO processing, and these observations have been confined only to males (Koehler et al. 2002; Dumont et al. 2009; Dumont and Payseur 2011a, 2011b; Baier et al. 2014; Peterson and Payseur 2021). Recent studies have shown that many wild-derived mouse strains from the *Mus musculus musculus* subspecies exhibit reversed patterns of heterochiasmy whereby males from the PWD/PhJ (PWD) strain make more class I COs than females, but curiously retain a shorter SC length like males from other strains (Peterson and Payseur 2021; Wooldridge et al. 2025). These unique qualities make PWD mice powerful tools for examining sex differences in the regulation of crossing over and the significance of chromosome axis length in heterochiasmy. To better understand the importance of chromosome axis length on sexually dimorphic CO frequency and patterning, we conducted an in-depth cytological analysis of CO distribution and SC length in five inbred mouse strains. We selected strains in which male class I CO frequencies (as measured by MLH1 foci) were low (DBA/2J [DBA] (Simon et al. 2025), CAST/EiJ [CAST] (Koehler et al. 2002; Dumont and Payseur 2011a; Baier et al. 2014)), intermediate (C57BL/6J [B6] (Koehler et al. 2002; Baier et al. 2014; Vrooman et al. 2014), 129S1/SvImJ [129S1] (Horan et al. 2018)), or high (PWD/PhJ [PWD] (Dumont and Payseur 2011a; Peterson and Payseur 2021)). This panel of strains was also chosen to capture a high degree of genetic diversity, comprising three subspecies (*M. m. domesticus* [DBA, 129S1, B6], *M. m. musculus* [PWD], and *M. m. castaneus* [CAST]) and four of the seven phylogenetic groups identified by Petkov et al. (2004). Divergence among these three subspecies began ∼130 to 420 thousand years ago (Phifer-Rixey et al. 2020), and despite a conserved karyotype consisting of 20 pairs of acrocentric chromosomes, F_1_ males hybrids of PWD × *M. m. domesticus* exhibit hybrid sterility driven by *Prdm9* (which directs DSB hotspots) and the hybrid sterility X-linked *Hstx2* locus (Flachs et al. 2012; Lustyk et al. 2019; Fotopulosova et al. 2024). In this study, we also performed a thorough analysis of sex differences in the trajectory of recombination from DSBs through COs in PWD and B6 mice. Our work reveals critical multifactorial sex and strain differences in CO regulation that disrupt the long-held understanding that sex differences in SC length are the predominant driver of mammalian heterochiasmy.

## Results

### Sex and strain differences in class I and II COs

**Figure 1:**
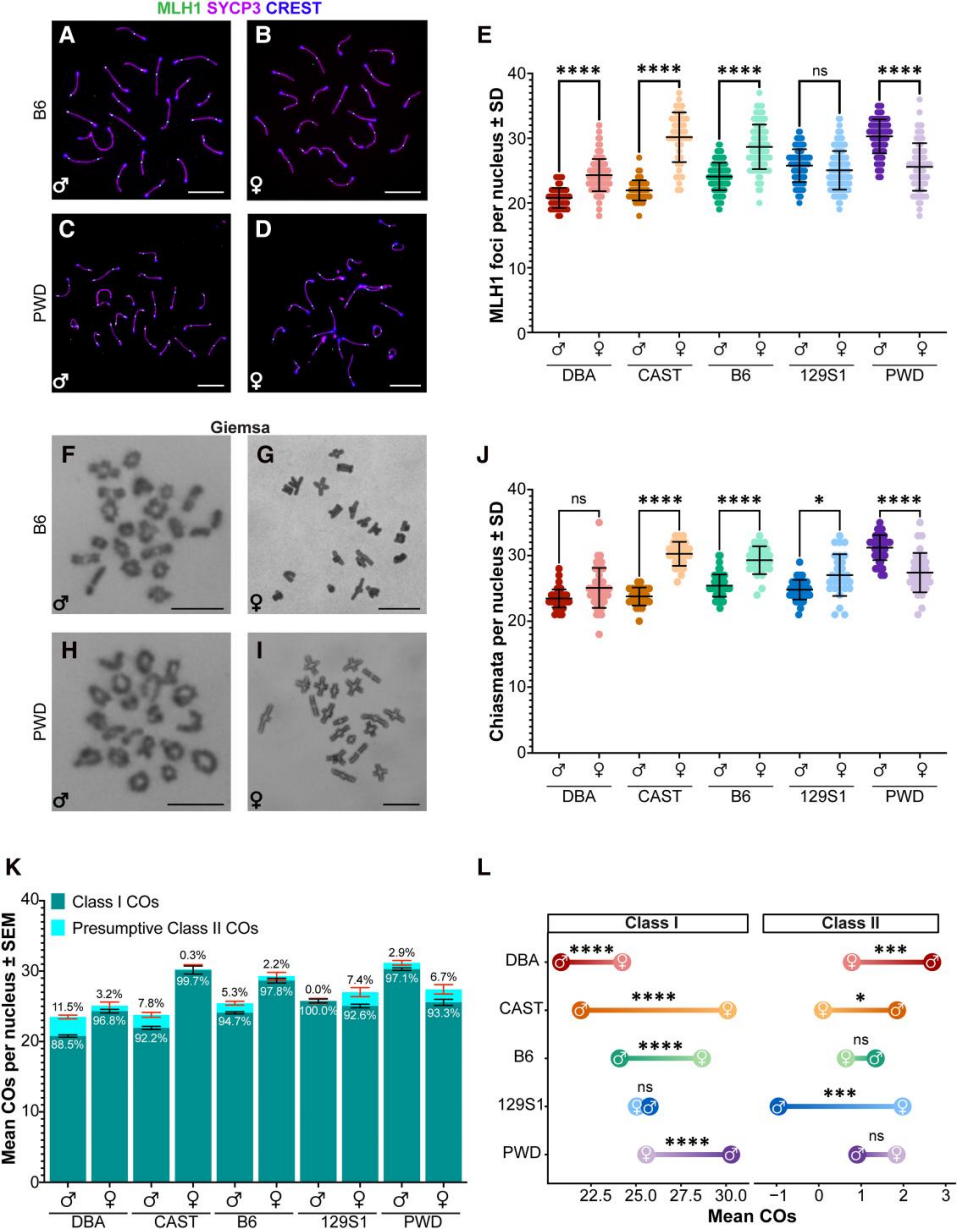
Patterns of sexual dimorphism in class I and II crossovers are inconsistent among diverse mouse strains. Representative immunofluorescence images of MLH1 (class I COs), SYCP3 (chromosome axes), and CREST (centromeres) in spermatocyte (a, c) and oocyte (b, d) chromosome spreads from B6 and PWD mice, respectively. e) Significant sex differences in mean MLH1 foci number were observed in DBA (red, *t* = 11.6), CAST (orange, *t* = 13.4), B6 (green, *t* = 12.4), and PWD (purple, *t* = 10.2), but not 129S1 (blue, *t* = 1.9) mice. Sample sizes (animals [cells]): DBA male (3 [86]), female (3 [94]); CAST male (3 [51]), female (3 [45]); B6 male (7 [200]), female (4 [104]); 129S1 male (3 [90]), female (4 [132]); and PWD male (7 [203]), female (3 [74]). Representative Giemsa-stained chiasmata spread from diakinesis/metaphase I spermatocytes (f, h) and oocytes (g, i) from B6 and PWD mice, respectively. j) Significant sex differences in mean chiasmata number were observed in CAST (*t* = 14.8), B6 (*t* = 8.3), 129S1 (*t* = 3.5), and PWD (*t* = 6.6) but not DBA (*t* = 3.2) mice. Sample sizes (animals [cells]): DBA male (3 [47]), female (9 [44]); CAST male (3 [28]), female (9 [27]); B6 male (3 [59]), female (4 [27]); 129S1 male (3 [44]), female (6 [29]); and PWD male (3 [52]), female (11 [34]). k) Presumptive class II CO numbers (light teal) were calculated by subtracting mean MLH1 foci (dark teal) from mean chiasmata for each sex and strain; class II CO standard error (red bars) was calculated by error propagation assuming independence between measures. l) Dumbbell plot of sex differences in CO pathway usage shows that the sex with more class I COs consistently has fewer class II COs, with significant sex differences in class II number in DBA, CAST, and 129S1 mice (male-female difference ± SE: 1.93 ± 0.58, *z* = 3.3; 1.75 ± 0.75, *z* = 2.33; and −2.94 ± 0.73, *z* = −4.01, respectively). Asterisks denote significant differences: ****P < 0.0001, ***P < 0.001, and *P < 0.05 (Games-Howell for e, j; Bonferroni for l). Scale bars represent 10 µm. Error bars represent SD (e, j) or SEM (k).

Prior comparisons of inter-strain sex differences in mouse CO frequency have used MLH1 foci as their only metric of COs, leaving critical questions about variation in the frequency of alternative CO pathways (e.g. the MUS81-EME1 mediated class II pathway) unanswered. To validate these previous findings and expand on them to examine total CO numbers, we used indirect immunofluorescence (IF) staining of MLH1 to assess class I COs in pachynema in both adult spermatocytes and fetal oocytes (Fig. 1a to d; Figure S1a and b, e and f, i and j, m and n, q and r). Chiasmata counts were obtained from Giemsa-stained diakinesis/prometaphase I adult spermatocytes and juvenile oocytes (Fig. 1f to i; Figure S1c and d, g and h, k and l, o and p, s and t).

For each sex and strain, the mean number of MLH1 foci per nucleus was quantified to estimate class I CO frequencies. One-way analysis of variance (ANOVA) indicated highly significant differences among groups (*P* < 0.0001; for full statistical comparisons, see Table S1). Females from most strains had significantly higher mean MLH1 foci counts by comparison to their respective males (Fig. 1e; Table 1). However, no significant sex difference in MLH1 foci was observed in 129S1 mice, while PWD mice exhibited an inverted sexual dimorphism with males averaging nearly five more MLH1 foci than females (*P* < 0.0001; Fig. 1e; Table 1, Table S1). Overall, PWD males had the highest mean MLH1 foci count of any sex and strain analyzed, while CAST mice showed the largest sex difference with females having 8.2 ± 0.6 more MLH1 foci than males (*P* < 0.0001; Fig. 1e; Table 1; Table S1). Chiasmata numbers generally corresponded to the strain-specific trends of sex-biased heterochiasmy we observed in MLH1 foci counts (*P* < 0.0001; Fig. 1j; Table 1; full statistical comparisons in Table S1). Notably, 129S1 females had more total chiasmata than their male counterparts (27.0 ± 0.59 and 24.82 ± 0.23, *P* < 0.05; Fig. 1j; Table 1; Table S1), making them the only strain to show a change in the directionality of heterochiasmy between male and female MLH1 and total CO counts.

**Table 1:** Crossover rates in male and female mice.

		Total COs			Class I COs			Class II COs	
Strain	Sex	No. of animals (cells)	Mean ± SEM	Mean diff. ± SE	No. of animals (cells)	Mean ± SEM	Mean diff. ± SE	Mean ± SEM	Mean diff. ± SE
DBA	Male	3 (47)	23.5 ± 0.2^G^	−1.6 ± 0.5	3 (86)	20.8 ± 0.2^G^	−3.5 ± 0.3	2.7 ± 0.3	1.9 ± 0.6
	Female	9 (44)	25.1 ± 0.5^D E G^		3 (94)	24.3 ± 0.3^D E^		0.8 ± 0.5	
CAST	Male	3 (28)	23.8 ± 0.3^F G^	−6.5 ± 0.4	3 (51)	21.9 ± 0.2^F^	−8.2 ± 0.6	1.9 ± 0.3	1.8 ± 0.8
	Female	9 (27)	30.3 ± 0.4^A B^		3 (45)	30.2 ± 0.6^A B^		0.1 ± 0.7	
B6	Male	3 (59)	25.4 ± 0.2^D E^	−3.9 ± 0.5	7 (200)	24.1 ± 0.2^E^	−4.6 ± 0.4	1.4 ± 0.3	0.7 ± 0.6
	Female	4 (27)	29.3 ± 0.4^B C^		4 (104)	28.7 ± 0.3^B^		0.6 ± 0.5	
129S1	Male	3 (44)	24.8 ± 0.2^E F^	−2.2 ± 0.6	3 (90)	25.8 ± 0.3^C^	0.7 ± 0.4	−1.0 ± 0.4	−2.9 ± 0.7
	Female	6 (29)	27.0 ± 0.6^C D^		4 (132)	25.1 ± 0.3^C E^		2.0 ± 0.6	
PWD	Male	3 (52)	31.2 ± 0.3^A^	3.8 ± 0.6	7 (203)	30.3 ± 0.2^A^	4.7 ± 0.5	0.9 ± 0.3	−0.9 ± 0.7
	Female	11 (34)	27.4 ± 0.5^C^		3 (74)	25.6 ± 0.4^C D^		1.8 ± 0.7	

Mean total and class I crossovers quantified using per-nucleus counts of chiasmata and MLH1 foci, respectively. Class II crossover numbers were calculated by subtracting mean MLH1 foci from mean chiasmata. Superscript letters within a column denote significant differences as determined by one-way ANOVA and Games-Howell post hoc test (*P* < 0.05); like letters indicate no difference.

Prior studies have demonstrated that a minority (∼5% to 10%) of COs in mice are MLH1-independent and are presumed to be produced via the class II pathway, mediated by MUS81-EME1 (Baker et al. 1996; Woods et al. 1999; Guillon et al. 2005; Holloway et al. 2008; Svetlanov et al. 2008). However, these studies were all conducted on the B6 background, and it is unknown whether proportions of class I and II COs are similar across mouse strains. Currently, a lack of commercially available antibodies viable for IF precludes direct cytological detection of class II COs. Thus, we estimated the frequency of class II COs by subtracting the mean number of MLH1 foci in pachynema from the total number of chiasmata (i.e. total CO number) and using the delta method to approximate their variance (Fig. 1k; Table 1). Within any given strain, the sex with the lower number of class I COs was estimated to have a higher relative number of class II COs (Fig. 1l). This trend held for all strains but only reached statistical significance for DBA (*P* < 0.001), CAST (*P* < 0.05), and 129S1 (*P* < 0.0001) mice (Fig. 1e; Table 1; Table S1). In the case of 129S1 males, the estimated number of class II COs was negative, highlighting that these values are estimates and should be interpreted with caution and underscoring the differences in quantitation methods represented by IF of surface spread chromosomes in prophase I and Giemsa staining of chromatin during diakinesis.

The low class I CO rates observed in CAST and DBA males (each approaching an average of 1 MLH1 focus per chromosome pair) raised the possibility that CO assurance may be more error-prone (i.e. less likely to make the obligate CO) in these mice. To test whether class II COs safeguard this obligate CO, we compared the percentage of pachytene cells containing at least one SC without an MLH1 focus (i.e. E0s; Figure S2a) to the percentage of diakinesis/prometaphase I cells with at least one prematurely separated homologous pair (i.e. univalents; Figure S2b). χ² analysis of the frequency of cells containing at least one E0 indicated highly significant differences among mouse strains (Χ^2^ = 69.0, *P* < 0.0001), and this occurrence was more common in females than males of the same strain (Figure S2c). Cells with univalent chromosomes were not observed in either CAST or DBA males despite having two of the highest incidences of E0s among males. Notably, the only example of univalent chromosomes in males was observed in the 129S1 strain, which our estimates suggest make few or no class II COs (Fig. 1k; Figure S2c). Taken together, these data suggest the class II pathway plays a critical role in ensuring that obligate COs are made on each chromosome pair and highlight the heterogeneous nature of class II pathway usage across divergent mouse strains.

### Sex differences in class I CO rates are not universally attributable to sex differences in SC length

**Figure 2:**
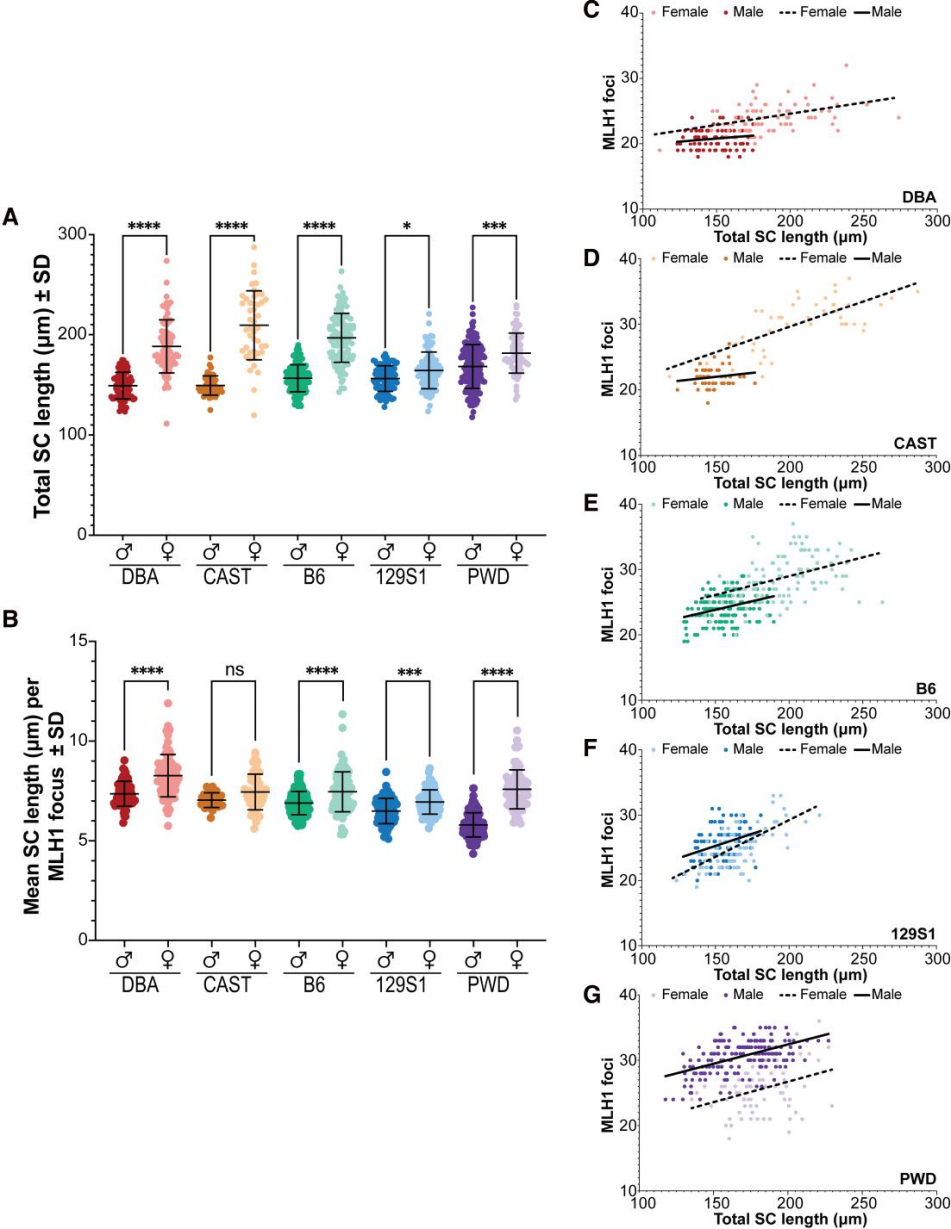
Sex differences in SC length do not universally explain sex differences in crossover rate. SC lengths and MLH1 foci position from the centromere were measured; samples sizes (animals [cells]): DBA male (3 [86]), female (3 [78]); CAST male (3 [51]), female (3 [45]); B6 male (7 [165]), female (4 [104]); 129S1 male (3 [90]), female (3 [86]); and PWD male (7 [173]), female (3 [74]). a) Females had significantly longer SCs than males in all mouse strains. b) Except for CAST mice (no sex difference), males had fewer microns of SC length per MLH1 focus. c to g) Multiple linear regression analysis of the effects of sex (male dark colors, female light colors; lines of best fit: male solid, female dashed) and total SC length on per-nucleus class I CO rates (MLH1 foci) for each strain: DBA, red (c); CAST, orange (d); B6, green (e); 129S1, blue (f); and PWD, purple (g). Asterisks denote significant differences: ****P < 0.0001, ***P < 0.001, and *P < 0.05 (one-way ANOVA with Games-Howell post hoc; a, b). Error bars represent SD.

Numerous studies using mouse models have shown compelling evidence to support two complementary hypotheses regarding the relationship between SC length and CO distribution: (i) cells with longer SCs make more COs (Lynn et al. 2002; Kleckner et al. 2003; Ruiz-Herrera et al. 2017; Wang et al. 2019) and (ii) higher CO frequencies in eutherian females are attributable to longer female SC length (Lynn et al. 2002; Tease and Hultén 2004; Gruhn et al. 2013; Wang et al. 2017). It is thus logical to predict SCs to be significantly longer in PWD males compared females; similarly, 129S1 males and females would likely show no significant difference in SC length. To test these hypotheses, we measured total SC lengths (μm) by tracing SYCP3. Because the X and Y chromosomes exclusively pair and undergo reciprocal exchange at the pseudoautosomal region, they do not reflect the broader CO landscape (Hinch et al. 2014; Acquaviva et al. 2020). We therefore measured all SCs in oocytes but only autosomal SCs in spermatocytes. Although this creates a slight female bias in the analysis of total SC length, the inclusion of an “extra” SC in the female data is not sufficient to change our ultimate conclusions. To standardize our data to account for the “extra” female SC, we also measured the microns of SC length per MLH1 focus on a per-chromosome basis (Fig. 2b).

Comparing mean SC lengths indicated highly significant differences among sexes and strains (*P* < 0.0001; see Table S1 for full statistical analysis). For all strains, females consistently had significantly longer SCs than their respective males (Fig. 2a; Table 2; Table S1). Although the magnitude of the sex difference was less extreme in PWD mice than in other strains, it was still highly significant (*P* < 0.001; Fig. 2a; Table S1). Across strains, mice with the highest MLH1 focus counts also had the longest total SC length for their respective sex (e.g. the longest vs. second longest SC lengths among males were PWD vs. B6 mice, mean ± SEM: 168.3 µm ± 1.7 and 156.6 µm ± 1.1, respectively; *P* < 0.0001; Fig. 2a; Table 2; Table S1). Our analysis of SC length per MLH1 focus indicated males had modestly, but significantly, fewer microns per focus by comparison to females (*P* < 0.0001; Fig. 2b; Table 2; see Table S1 for full statistical comparison). Regardless of sex, most mice had between 7.0 and 7.5 μm/focus (Fig. 2b). However, CAST females and PWD males were notable outliers (8.27 ± 0.12 μm/focus and 5.86 ± 0.05 μm/focus, respectively), with PWD mice showing the highest sex difference (males averaging 1.78 μm/focus less than females, *P* < 0.0001; Table 2; Table S1).

**Table 2:** Total SC length per nucleus and microns of SC per MLH1 focus.

		Early pachynema		Mid-pachynema		
Strain	Sex	No. of animals (cells)	Mean SC length (µm) ± SEM	No. of animals (cells)	Mean SC length (µm) ± SEM	Mean µm SC/MLH1 focus ± SEM
DBA	Male	—	—	3 (86)	149.2 ± 1.43^F^	7.36 ± 0.08^BC^
	Female	—	—	3 (78)	188.3 ± 3.00^BC^	8.27 ± 0.12^A^
CAST	Male	—	—	3 (51)	149.3 ± 1.34^F^	7.04 ± 0.07^CD^
	Female	—	—	3 (45)	209.5 ± 5.14^A^	7.45 ± 0.13^BC^
B6	Male	4 (79)	130.0 ± 1.0^D^	7 (165)	156.6 ± 1.05^E^	6.90 ± 0.05^D^
	Female	4 (67)	226.0 ± 4.1^A^	4 (104)	196.8 ± 2.39^AB^	7.46 ± 0.10^B^
129S1	Male	—	—	3 (90)	156.2 ± 1.35^E^	6.50 ± 0.07^E^
	Female	—	—	3 (86)	164.4 ± 1.97^D^	6.95 ± 0.07^D^
PWD	Male	5 (72)	140.1 ± 1.3^C^	7 (173)	168.3 ± 1.66^D^	5.80 ± 0.05^F^
	Female	8 (64)	205.1 ± 4.6^B^	3 (74)	181.6 ± 2.32^C^	7.58 ± 0.11^B^

Mean SC length represents total length of all SCs per nucleus. Mean microns per MLH1 focus were calculated on a per-SC basis. Superscript letters within a column denote significant differences as determined by one-way ANOVA and Games-Howell post hoc test (*P* < 0.05); like letters indicate no difference. — denotes group analysis was not performed.

To determine the relative effects of sex (female as the reference group) and SC length on MLH1 foci count, we performed independent multivariable linear regression analyses for each strain (Fig. 2c to g; Table S2). For all strains, the inclusion of a sex-SC length interaction did not significantly improve the model's fit, indicating the effect of SC length on MLH1 foci was similar between males and females within each strain. The multiple regression was highly significant and explained ∼50% (i.e. adj. *R*^2^ = 0.5) of the variation in MLH1 focus numbers for most strains (Fig. 2c, e, g; Table S2). In contrast, the regression model showed very strong fit for CAST mice (explaining 80% of the variation in MLH1 focus numbers; Fig. 2d), but a more moderate level of fit for 129S1 mice (explaining 32% of variation; Fig. 2f; Table S2). Thus, sex and SC length account for much of the variation in MLH1 focus numbers in most mouse strains, but they are less consequential (although still significant) factors in 129S1 mice.

For all strains, both SC length and sex were strong, highly significant predictors of MLH1 foci number; however, the magnitude and direction of their standardized effect sizes (β) varied considerably (Table S2). Consistent with prior studies (Lynn et al. 2002; Kleckner et al. 2003; Wang et al. 2019), SC length universally had a positive effect on MLH1 foci number. The effect of sex on MLH1 focus numbers was also consistent with our observations of sex differences in CO frequency (Fig. 1e), showing a negative effect of male sex among DBA, CAST, and B6 mice (Fig. 2c to e), but a positive effect for 129S1 and PWD mice (Fig. 2f, g; Table S2). For CAST, B6, and 129S1 mice, SC length had a larger effect on MLH1 foci count than did sex; this difference was particularly striking in 129S1 mice, where β for SC length was 2-fold larger than β for sex (Fig. 2d to f; Table S2). By contrast, for DBA and PWD mice, changes in MLH1 focus counts were attributable more to sex effects than SC length. For DBA mice, this difference was subtle (1.2-fold increase over SC length; Fig. 2c), but the sex effect was much more pronounced in PWD mice (2-fold increase over SC length; Fig. 2g; Table S2).

Although the interaction of sex and SC length was not predicted to significantly affect the regression model for any strain, sex and SC length did show moderate collinearity in DBA, CAST, and B6 mice (variance inflation factor [VIF] = 1.91, *R*^2^ = 0.48 for DBA mice; VIF = 2.52, *R*^2^ = 0.60 for CAST mice, and VIF = 2.13, *R*^2^ = 0.53 for B6 mice). In contrast, there was nearly no collinearity in 129S1 and PWD mice (VIF = 1.07, *R*^2^ = 0.06, VIF = 1.08, *R*^2^ = 0.08, respectively), suggesting that SC length and sex have nearly independent effects on MLH1 foci number in these strains. Taken together, this model suggests that, in contrast to strains like B6 and CAST, PWD mice do not exhibit parallel sexual dimorphism in SC length and class I COs, with males making more COs despite a shorter axis. Further, the assumption that sex mediates the effect of SC length on MLH1 foci number is not statistically supported in our analysis of PWD and 129S1 mouse strains.

### Males have stronger CO interference than females

A weaker CO interference resulting in narrower placement of adjacent MLH1 foci could provide an explanation for the exceptional sexual dimorphism in PWD mouse CO rates. To test this hypothesis, we analyzed three related parameters of interference: (i) distribution of MLH1 foci along the length of the SC, (ii) the distance between adjacent MLH1 foci, and (iii) the evenness of MLH1 distribution.

#### Distribution of MLH1 foci

**Figure 3:**
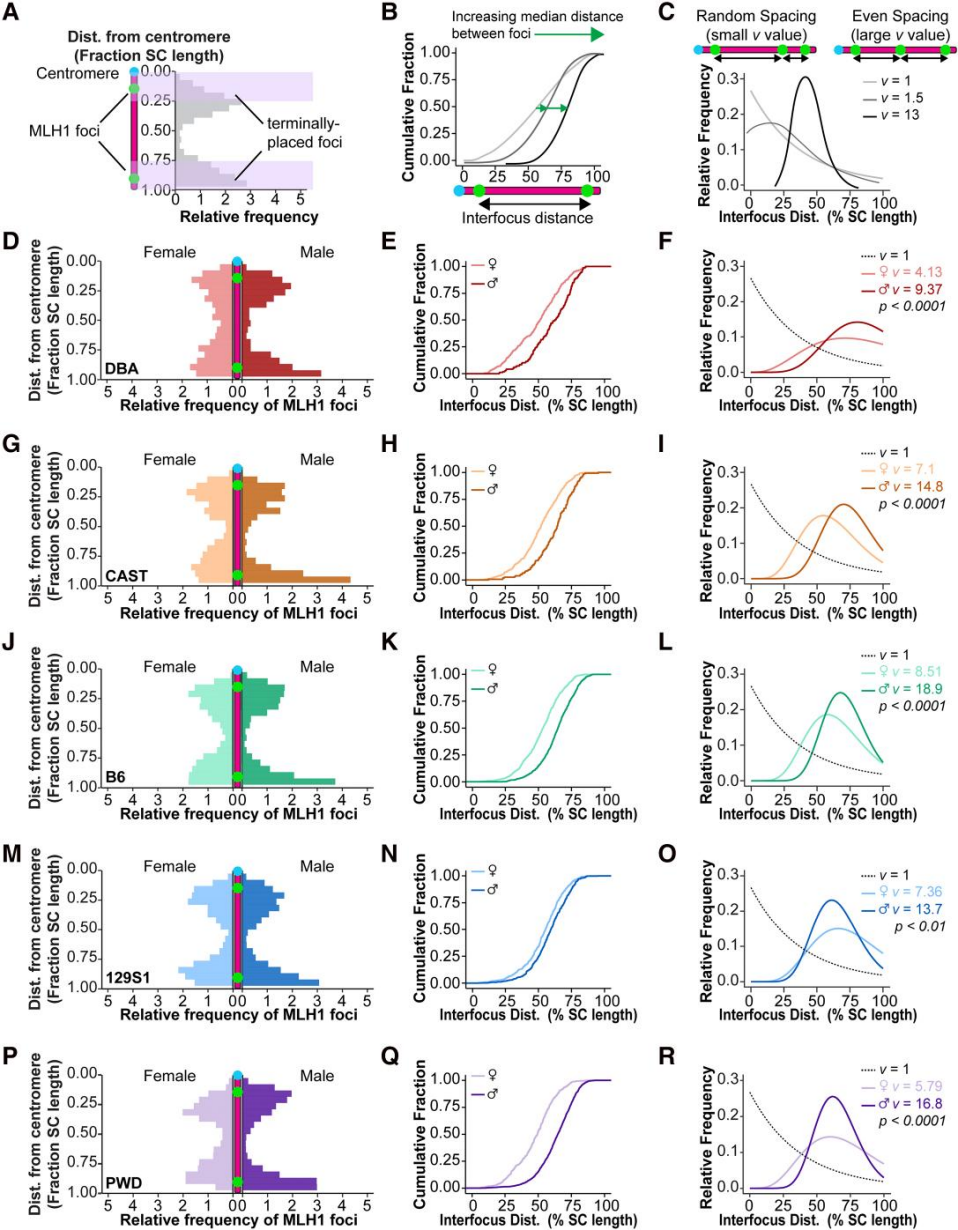
Males exhibit stronger crossover interference. Three parameters were used to analyze CO interference strength: terminal placement of MLH1 foci (d, g, j, m, p), inter-focus distance standardized by %SC length (e, h, k, n, q), and evenness of MLH1 foci spacing along the SC (f, i, l, o, r); interpretation overviews are shown in a) to c), respectively. a) The *y* axis represents fractional SC length from centromere (0.0) to distal telomere (1.0); purple bars denote terminal SC ends (0.0 to 0.25 and 0.75 to 1.0). Histograms show relative frequency of MLH1 foci at each centromere distance. Males (dark bars) had significantly more terminally placed foci than females (light bars; Fisher's exact test): 72.9% vs. 59.7% in DBA (d, OR = 0.55, *P* < 0.0001), 72.1% vs. 59.8% in CAST (g, OR = 0.58, *P* < 0.001), 72.9% vs. 61.8% in B6 (j, OR = 0.60, *P* < 0.0001), and 76.2% vs. 52.6% in PWD (p, OR = 0.35, *P* < 0.0001), but not in 129S1 (67.6% vs. 64.8%, OR = 0.88, *P* = 0.2). Among females, PWD mice had less terminally placed foci were than B6 (OR = 1.46, *P* < 0.001) and 129S1 (OR = 1.66, *P* < 0.0001). Among males, 129S1 mice had significantly fewer terminally placed foci than PWD (OR = 0.65, *P* < 0.0001). b) ECDF *x* axis represents standardized inter-focus distance (%SC length); *y* axis represents the fraction of SCs with inter-focus distance at or below the corresponding *x*-value. Steeper curves indicate tighter clustering; rightward shifts indicate greater median distances. Males had significantly greater median inter-focus distances than females (KS with Bonferroni correction; Table S4) in DBA (e, KS = 0.26, *P* < 0.0001), CAST (h, KS = 0.31, *P* < 0.0001), B6 (k, KS = 0.34, *P* < 0.0001), 129S1 (n, KS = 0.13, *P* < 0.05), and PWD (q, KS = 0.45, *P* < 0.0001). c) Standardized inter-focus distances (%SC length) for each SC were fit to the gamma distribution using shape parameter (*v*). Larger *v* values indicate more symmetrical, bell-shaped distributions and thus more regular MLH1 spacing (stronger interference); *v* = 1 (dotted line) represents an exponential distribution indicating random spacing and no interference. Males had significantly larger v than females in all strains: DBA (f) LR = 50.16; CAST (i) LR = 125.29; B6 (l) LR = 200.93; 129S1 (o) LR = 9.29; and PWD (r) LR = 29.87.

To examine the sex- and strain-specific effects of interference on MLH1 distribution on SCs with two foci, we normalized measurements of MLH1 foci positions (as a fraction of SC length), beginning from the centromere. We then compared the likelihood that MLH1 foci were positioned in the terminal ends (i.e. <0.25 or >0.75) vs. the central 50% of the SC (Fig. 3a). Across sexes and strains, the two MLH1 foci exhibited bimodal distribution, pushing placement of each focus toward opposing ends of the SC (Fig. 3d, g, j, m, p). Fisher's exact test indicated that for all strains except 129S1 mice, males were significantly more likely than their corresponding females to place MLH1 foci in the terminal quartiles of the SC. Mice of the same sex were overall similar to each other across strains with two notable exceptions. Among females, PWD mice were the least likely to have terminal MLH1 foci, significantly less likely than B6 (*P* < 0.001) and 129S1 (*P* < 0.0001) mice. Among males, 129S1 mice were the least likely to have terminal foci, significantly less likely than PWD males (*P* < 0.0001).

To determine if chromosomes behaved differently based on their relative lengths, we also categorized SCs: the five longest (lower 25%; Figure S4a) and five shortest (upper 25%; Figure S4c). Long SCs showed the same trends found among pooled SCs (described above). Short SCs showed similar foci distributions regardless of genetic background, with no significant differences observed among males (Χ^2^ = 6.5, *P* = 0.16) or females (Χ^2^ = 7.1, *P* = 0.13) across strains. Females from most strains showed an increasing likelihood of terminal foci placement among short SCs relative to long SCs, while males showed similar foci distribution regardless of SC length (Figure S4a, c). These trends erased sexual dimorphism of MLH1 distribution on short SCs in all strains except PWD mice (75.7% of foci were terminally placed in males vs. 55.6% in females, *P* < 0.0001; Figure S4c). However, short SCs with multiple MLH1 foci were too scarce in CAST and DBA males to reliably assess sex differences in those strains (Figure S4c).

#### Distance between MLH1 foci

To test whether the spacing between adjacent class I COs was greater in one sex, we examined the normalized distance (percentage of SC length) between MLH1 foci. The cumulative fraction of inter-focus distances was plotted (Fig. 3b) and significant differences between groups determined using Kolmogorov-Smirnov (KS) tests with a Bonferroni correction for multiple comparisons (full statistical analysis in Table S4). For all mouse strains, males showed a significantly larger median spacing between adjacent MLH1 foci by comparison to females (Fig. 3e, h, k, n; Table S4). This difference was greatest in PWD mice (male vs. female inter-focus distance of 64.7% vs. 49.3%, *P* < 0.0001; Fig. 3q; Table S4). Across strains, males had similar inter-focus spacing except for 129S1 mice, which had significantly closer-spaced foci (59.40%) than either B6 (64.76%, *P* < 0.0001) or PWD males (*P* < 0.0001; Table S4). Greater inter-strain variance was observed among females, with DBA (51.27%) and PWD (49.32%) mice having significantly narrower spacing by comparison to either 129S1 (55.39%; *P* < 0.01) or B6 (53.50%; *P* < 0.01) mice (Table S4). Consistent with our observations in MLH1 distribution, the longest five SCs per cell showed similar trends to the pooled analysis of inter-focus distances with males spacing foci significantly further apart than females (Figure S4b). In contrast, MLH1 spacing on short SCs was similar across all sexes and strains except for PWD mice, where males again showed wider spacing between foci than females (63.2% vs. 47.0%, *P* < 0.001; Figure S4d; Table S4).

#### Evenness of MLH1 distribution

The evenness of MLH1 spacing can be represented by the shape parameter (*v*) of distances between adjacent foci fitted to the gamma distribution, with larger values of *v* representing more symmetrical distributions and thus stronger interference (Fig. 3c). Because this analysis only accounts for chromosomes with multiple MLH1 foci, we employed statistical censoring to incorporate chromosomes with only one MLH1 focus in our analysis of interference. Measuring MLH1 distances from the centromere, the distance from the last (or only) focus to the end of the SC was considered right-censored while inter-focus distances were non-censored. Comparing the fitted gamma distributions between sexes for each strain using likelihood ratio tests indicated highly significant sex differences, with males exhibiting larger shape parameters (*v*)—and thus more even foci distributions—by comparison to females (Fig. 3f, i, l, o, r).

As most of our analyses of CO interference relied on SCs with at least two MLH1 foci, we wanted to determine if the frequency of SCs with multiple foci differed among sexes and strains. Of the five strains analyzed, only PWD males had multiple MLH1 foci on more than half their chromosomes (Figure S3b). Short SCs with multiple class I COs were uncommon, accounting for less than 10% of short SCs for most groups. However, their frequency among PWD males was almost twice that of PWD females (16.5% vs. 8.4%) and nearly four times that of B6 males (4.4%; Figure S3b). To estimate the likelihood of having multiple MLH1 foci on SCs at different lengths, we performed a logistic regression using SC length, sex, and strains, along with all two- and three-way interactions as predictors (Figure S3a, c; Table S3). This model indicated that for DBA and CAST mice, females were significantly more likely to have multiple MLH1 foci on short (5 µm) SCs (*P* < 0.0001 for both strains; Figure S3a, c) while males were more likely on long (15 µm) SCs (*P* < 0.01 for DBA mice, *P* < 0.05 for CAST mice; Figure S3a, c). Although B6 mice showed a similar trend, sex differences did not reach statistical significance at any SC length. Notably, only PWD males were significantly more likely than their corresponding females to have multiple MLH1 foci at all SC lengths (*P* < 0.0001; Figure S3a, c).

Our data demonstrate that the evenness and spacing between MLH1 foci is greater in males than in females, leading chromosomes with multiple class I COs to have more terminally positioned COs in males than females. Taken together, this suggests that CO interference is stronger in males than in females, supporting previous findings (Petkov et al. 2007; Morgan 2026). Consistently, PWD mice showed the most striking sex differences in MLH1 distribution and spacing. Although PWD males did trend toward having the highest likelihood of terminally positioned foci and the widest spacing between foci, this increased tendency was not significantly higher than in B6 males. By contrast, PWD females consistently exhibited more central MLH1 placement with narrower inter-focus spacing. Thus, the large sex difference in PWD MLH1 distribution was more likely an effect of females positioning a higher proportion of foci closer together and further from the SC ends. Thus, we reject our hypothesis that weaker interference in male mice explains the atypical sexual dimorphism of the PWD CO landscape. Instead, male PWD mice paradoxically have more class I COs spaced further apart on shorter axes by comparison to their female counterparts. Further, PWD males reliably make multiple class I COs, even on their shortest SCs, despite these limitations.

### Strong suppression of pericentromeric COs in PWD females

**Figure 4:**
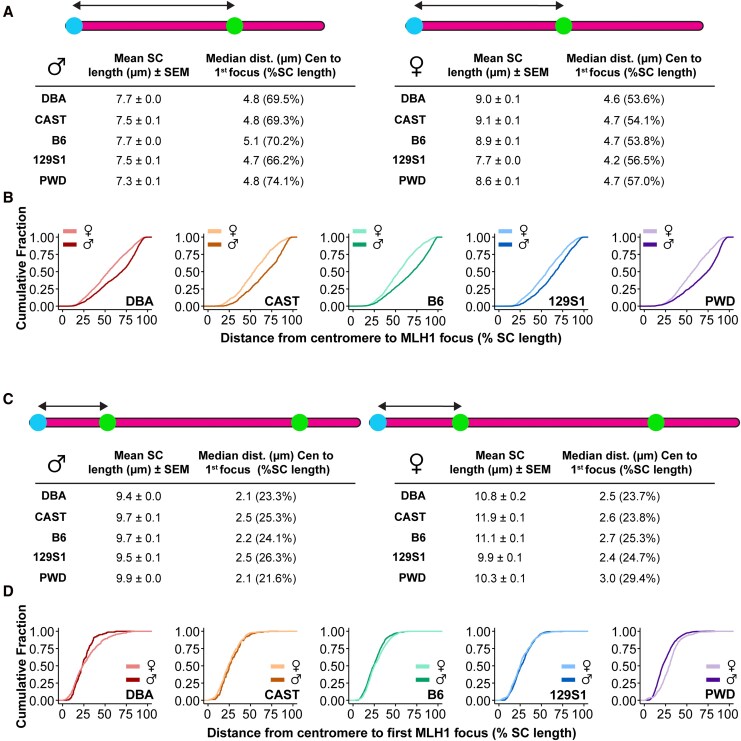
PWD males exhibit weaker suppression of pericentromeric crossovers. Pericentromeric CO suppression was measured as the normalized distance (%SC length) from the centromere to the first MLH1 focus for SCs with one (a, b) or multiple (c, d) MLH1 foci. a and c) Diagrams illustrate the median distance (black double arrow) between the centromere (blue) and the first MLH1 focus (green) along the SC (magenta), with mean SC lengths shown. b and d) ECDF *x* axis represents standardized distance (%SC length) from centromere to first MLH1; *y* axis represents the fraction of SCs with centromere-MLH1 distance at or below the corresponding *x*-value. Steeper curves indicate tighter clustering; rightward shifts indicate greater median distance. b) For SCs with one MLH1 focus, males consistently had greater centromere distances than females in all strains (Kolmogorov-Smirnov: DBA, KS = 0.21, *P* < 0.0001; CAST, KS = 0.26, *P* < 0.0001; B6, KS = 0.27, *P* < 0.0001; 129S1, KS = 0.17, *P* < 0.0001; PWD, KS = 0.27, *P* < 0.0001). d) For SCs with multiple MLH1 foci, significant sex differences in centromere-MLH1 distance were only observed in PWD (KS = 0.26, *P* < 0.0001).

Pericentromeric suppression of crossing over is a well-documented phenomenon in meiosis (Nambiar and Smith 2016; Kuhl and Vader 2019; Pazhayam et al. 2021) and is clearly observable in our MLH1 distribution data as a near absence of MLH1 across groups in the interval nearest the centromere (Fig. 3d, g, j, m, p). To test whether pericentromeric CO suppression differs among sexes and strains, we compared the normalized distance (%SC length) from the centromere to the first MLH1 focus (Fig. 4; full statistical analysis in Table S5). As described earlier, we were confined to exploring centromere effects on class I COs (MLH1 foci) because of the absence of reliable class II CO markers. Regardless of background, chromosomes with one MLH1 focus consistently exhibited significantly more telocentric MLH1 positioning in males by comparison to females (Fig. 4a and b). Across strains, PWD males and females tended to place their foci farther from the centromere than other strains (74.06% and 56.97% of the SC length, respectively), with PWD males showing significantly further placements than DBA (69.49%, *P* < 0.0001) or 129S1 (66.22%, *P* < 0.0001) males (Fig. 4a; Table S5). However, we reasoned that chromosomes with multiple MLH1 foci would best reveal the minimal distance of pericentromeric suppression because the centromere effect (pushing COs to the distal telomere) would be in opposition to interference (pushing COs toward chromosome ends). For chromosomes with multiple MLH1 foci, sex differences among strains largely vanished with the sole exception of PWD mice (21.60% vs. 29.43%, for males and females, respectively, *P* < 0.0001; Fig. 4c and d; Table S5). Across strains, PWD males position their first MLH1 focus closest to the centromere, but the difference was only significant compared against 129S1 males (*P* < 0.0001; Table S5). In contrast, the first MLH1 focus was spaced significantly farther from the centromere in PWD females than in all other strains (Table S5). These results indicate that the striking difference in centromere effect in PWD mice is primarily a result of stronger pericentromeric suppression of class I COs in PWD females.

### Sexually dimorphic dynamics of DSB repair differ between B6 and PWD mice

Having identified significant and unexpected sex differences in the PWD mouse CO landscape, we next examined the localization dynamics of proteins essential for DSB repair events leading to the formation of class I COs in order to ask whether specific steps in the pathway might show divergence between males and females. For the remainder of the studies described in this manuscript, we focused our analysis on PWD mice and use the well-characterized B6 mouse strain as a reference group.

**Figure 5:**
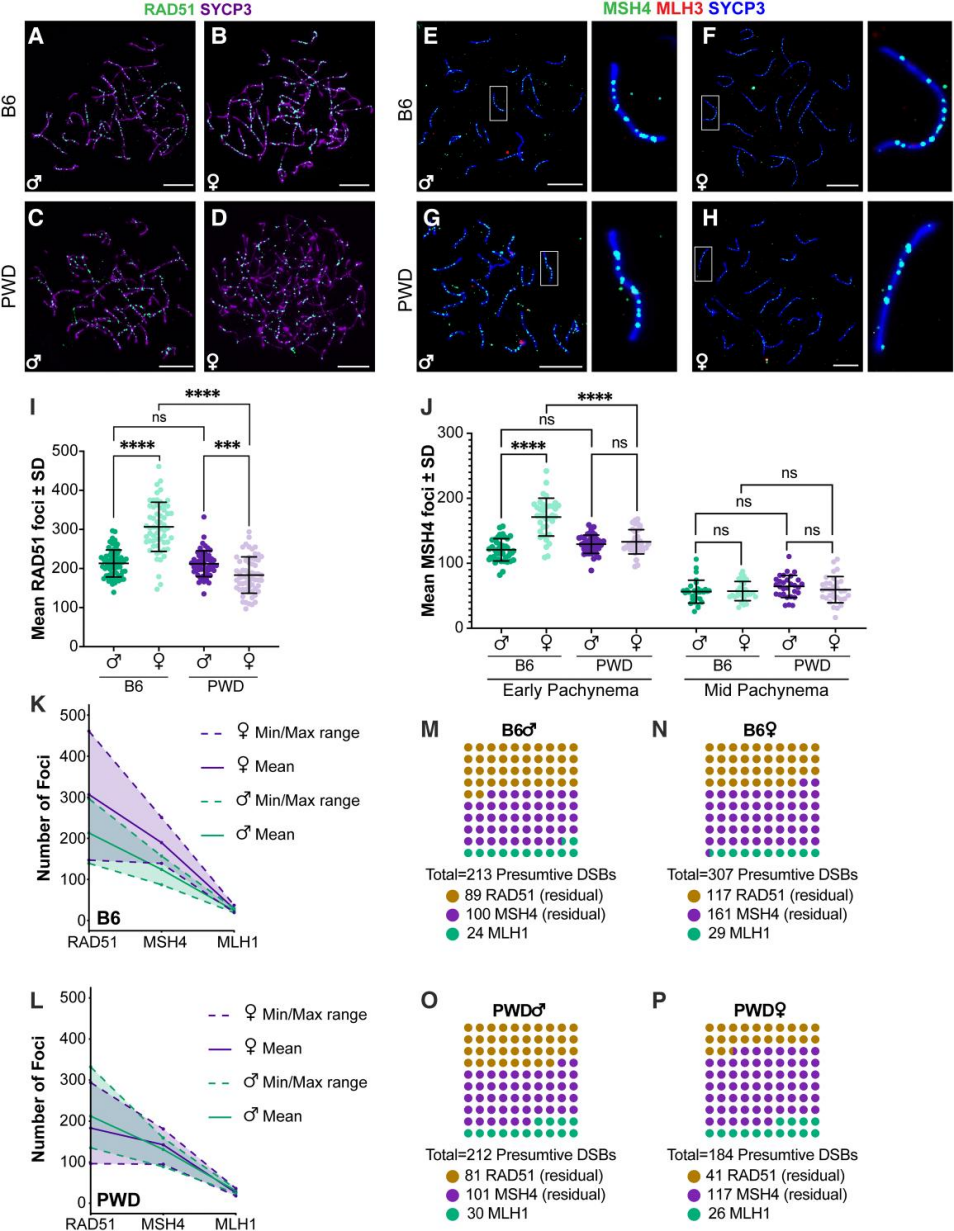
PWD and B6 mice exhibit different sex-specific dynamics in the regulation of crossover designation. Representative immunofluorescence images of zygotene spermatocytes and oocytes from B6 (a, b) and PWD (c, d) mice stained for RAD51 (green, proxy for presumed DSBs) and SYCP3 (magenta). Representative early pachytene spermatocytes and oocytes from B6 (e, f) and PWD (g, h) mice stained for MSH4 (green, proxy for CO intermediates), MLH3 (red, class I COs), and SYCP3 (blue). Enlarged views of SCs within white boxes are shown at right. i) Comparison of mean RAD51 foci number (ANOVA *F* = 83.09, *P* < 0.0001) indicates significant sex differences in B6 (*t* = 10.2) and PWD (*t* = 3.9); samples sizes (animals [cells]): B6 male (4 [65]), female (3 [60]); PWD male (3 [56]), female (3 [61]). j) Comparison of mean MSH4 foci number in early (ANOVA *F* = 43.71, *P* < 0.0001) and mid-pachynema (*F* = 1.3, *P* = 0.3) indicated significant sex differences only in B6 at early pachynema (*t* = 9.12) samples sizes (animals [cells]): for early pachynema: B6 male (3 [43]), female (4 [36]); PWD male (4 [42]), female (6 [36]); for mid-pachynema: B6 male (3 [29]), female (3 [31]); PWD male (3 [30]), female (3 [32]). k and l) Progressive reduction from RAD51 (presumptive DSBs) to MSH4 (CO licensed sites) to MLH1 (designated class I COs) in male (green) and female (purple) B6 (k) and PWD (l) mice. Solid lines indicate mean foci number; dotted lines indicate minimum and maximum observed. m to p) Presumptive DSB number (circles) and proportion of DSBs resolved as class I COs (green), converted to MSH4-marked licensed sites but not designated as COs (purple), or repaired by NCO pathways (orange) for B6 (m, n) and PWD (o, p) males and females, respectively. Asterisks denote significant differences: ****P < 0.0001, ***P < 0.001 (Games-Howell post hoc; i, j). Scale bars represent 10 µm. Error bars represent SD.

Previous studies have suggested that strain differences in genome-wide CO rates are determined long before CO designation, with differences evident in the number of induced DSBs at the onset of meiosis (Baier et al. 2014; Peterson and Payseur 2021). To determine whether these observations held for males vs. females and between B6 and PWD mice, we used indirect IF staining against the RecA homolog, RAD51, which binds to the resected ends of all induced DSBs in zygonema to initialize homologous recombination (Fig. 5a to d) (Lao et al. 2013; Brown and Bishop 2014; Hinch et al. 2020). Analysis of mean counts of RAD51 foci indicated highly significant differences among groups (*P* < 0.0001). Surprisingly, males of each strain did not significantly differ in RAD51 foci number (mean ± SEM: 213 ± 4.3 and 212.1 ± 4.4 for B6 and PWD, respectively; *P* = 1.0; Table 3; Fig. 5i). However, each strain exhibited opposing sexual dimorphisms: In B6 mice, females had considerably more RAD51 foci by comparison to males (306.6 ± 8.1, *P* < 0.0001), while PWD females had fewer than their respective males (183.1 ± 5.9, *P* < 0.001; Table 3; Fig. 5i). These data suggest that sex differences in crossing over for these strains might begin prior to or at the onset of DSB induction.

**Table 3:** Mean foci number of RAD51, MSH4, and HEI10 foci throughout prophase I.

		B6		PWD	
		Male	Female	Male	Female
Zygonema	Number of animals (cells)	4 (65)	3 (60)	3 (56)	3 (61)
	Mean RAD51 foci ± SEM	213.0 ± 4.3^B^	306.6 ± 8.1^A^	212.1 ± 4.4^B^	183.1 ± 5.9^C^
Early pachynema	Number of animals (cells)	3 (43)	4 (36)	4 (42)	6 (36)
	Mean MSH4 foci ± SEM	120.8 ± 2.6^C^	171.1 ± 4.9^A^	129.4 ± 2.2^BC^	133.1 ± 3.1^B^
	Mean µm SC/MSH4 focus ± SEM	1.17 ± 0.03^A^	1.42 ± 0.05^B^	1.12 ± 0.02^A^	1.75 ± 0.06^C^
	Number of animals (cells)	3 (36)	3 (31)	3 (29)	4 (28)
	Mean HEI10 foci ± SEM	18.9 ± 1.5^C^	96.2 ± 4.0^A^	45.1 ± 3.4^B^	81.6 ± 3.8^A^
	Mean µm SC/HEI10 focus ± SEM	5.52 ± 0.16^A^	2.90 ± 0.13^C^	3.88 ± 0.19^B^	3.12 ± 0.13^C^
Mid-pachynema	Number of animals (cells)	3 (29)	3 (31)	3 (30)	3 (32)
	Mean MSH4 foci ± SEM	54.7 ± 3.2	55.4 ± 2.6	62.4 ± 3.0	57.6 ± 3.4
	Mean µm SC/MSH4 focus ± SEM	3.36 ± 0.20^BC^	4.46 ± 0.16^A^	2.94 ± 0.20^C^	4.11 ± 0.22^AB^
	Number of animals (cells)	3 (35)	3 (29)	3 (31)	3 (30)
	Mean HEI10 foci ± SEM	26.3 ± 0.7^B^	39.8 ± 2.6^A^	33.3 ± 0.7^A^	37.4 ± 1.5^A^
	Mean µm SC/HEI10 focus ± SEM	6.41 ± 0.12^A^	5.77 ± 0.23^AB^	5.19 ± 0.13^B^	5.56 ± 0.19^B^

Mean foci numbers were assessed per nucleus while mean microns per MSH4 or HEI10 focus were calculated on a per-SC basis. Superscript letters within a row denote significant differences as determined by one-way ANOVA and Games-Howell post hoc test (*P* < 0.05); like letters indicate no difference.

We next used indirect IF staining of the DNA mismatch repair protein MSH4 as a marker of CO licensing (Fig. 5e to h; Figure S5a to d). Although peak MSH4 foci number has been reported to occur in late zygotene mouse spermatocytes (Cole et al. 2012), we chose to analyze MSH4 in early pachynema in order to normalize counts by SC length in order to further examine the association between chromosome axis length differences and CO formation (Figure S5e; Table 3). Comparison of mean total MSH4 foci number indicated highly significant differences (*P* < 0.0001). In B6 mice, females had significantly more foci than males (mean ± SEM: 171.1 ± 4.7 and 120.8 ± 2.6, respectively, *P* < 0.0001); however, in PWD mice, there was no significant sex difference (129.4 ± 2.2 and 133.1 ± 3.1 for males and females, respectively, *P* = 0.9; Table 3; Fig. 5j). Sex differences in SC length were more pronounced in early pachynema than in mid, with females averaging 60 to 100 µm longer than males (Table 2; Figure S6b). When MSH4 foci counts were weighted by SC length, clear sex differences in foci density were observed in both strains, with females consistently having more microns SC per focus by comparison to males (Table 3; Figure S5e). By either metric (total MSH4 foci or weighted by SC length), males from each strain showed no significant difference.

We additionally analyzed MSH4 foci number in mid-pachynema (Figure S5a to d; Table 3). As pachynema progresses, MSH4 foci decline with an increasing proportion showing colocalization with class I CO markers while residual MSH4 is depleted as a consequence of NCO DNA repair (Kneitz et al. 2000; Lenzi et al. 2005; Storlazzi et al. 2010; Cole et al. 2012). As expected, MSH4 foci were less numerous in mid-pachynema than at earlier stages, with all groups averaging from 54 to 63 ± 4 foci per nucleus. There was no significant difference between PWD and B6 males or females in total foci number (Fig. 5j; Table 3), but when weighting foci by SC length, males from each strain showed significantly higher densities of MSH4 than their corresponding females (Table 3; Figure S5e). The lack of a significant difference between males of different backgrounds is suggestive of strain differences in the proportion of residual MSH4 foci in mid-pachynema. To test this, we examined the colocalization of MSH4 and the class I CO marker, MLH3, finding significant sex and strain differences. B6 females showed higher proportion of MSH4 foci colocalized with MLH3 than did their male counterparts (50.6% and 42.1%, respectively, *P* < 0.0001; Figure S5f), but PWD mice exhibited the inverse sexual dimorphism (males vs. females: 47.9% and 41.7%, *P* < 0.001; Figure S5f).

Our analysis of mean RAD51, MSH4, and MLH1 foci numbers makes it possible to examine the overall trajectory of the class I CO pathway in males and females (Fig. 5k and l). To compare efficiency of establishing CO intermediates, we examined the ratios of MSH4 to RAD51 foci as a proxy for licensing efficiency using generalized linear mixed models (Fig. 5m to p). B6 males and females had similar ratios (0.57 and 0.56, respectively), but there was a much larger difference in PWD males and females (0.61 and 0.73). Comparison of the log-transformed ratios by generalized linear mixed modeling indicated that a significantly larger proportion of DSBs progress to CO intermediates in PWD females than in either PWD males (*z* = 3.02, *P* < 0.05) or B6 females (*z* = −4.41, *P* < 0.0001). No significant differences were observed among the other groups. Applying the same approach to MLH1:RAD51 as an estimate of the economical conversion of DSBs to mature class I COs, we found PWD mice had significantly larger proportions of DSBs resolve as class I COs for both males (0.14 and 0.11 in PWD and B6 mice, respectively; *z* = −5.76, *P* < 0.0001) and females (0.14 and 0.09 in PWD and B6 mice, respectively; *z* = −8.05, *P* < 0.0001). Within strains, only B6 mice showed a significant sex difference with males making more class I COs per DSB than females (*z* = −4.45, *P* < 0.0001). Finally, to estimate CO designation efficiency, we compared MLH1:MSH4, finding highly significant sex and strain effects. Males of both strains consistently had larger ratios than their corresponding females (B6 males vs. females: 0.20 and 0.17, *z* = −4.14, *P* < 0.001; PWD males vs. females: 0.23 and 0.19, *z* = −4.53, *P* < 0.0001), and ratios in PWD mice were consistently larger than in B6 mice (males: *z* = −3.93, *P* < 0.001; females: *z* = −2.66, *P* < 0.05). Taken together, these data suggest critical sex and strain differences in regulation and efficiency at multiple steps of crossing over: B6 and PWD mice exhibit opposite sexual dimorphism in DSB induction; sex differences in CO licensing efficiency are specific to PWD mice (females more efficient than males); but both strains show more efficient CO designation in males than in females.

### Sex- and strain-specific accumulation of the pro-CO factor, HEI10, in pachynema

**Figure 6:**
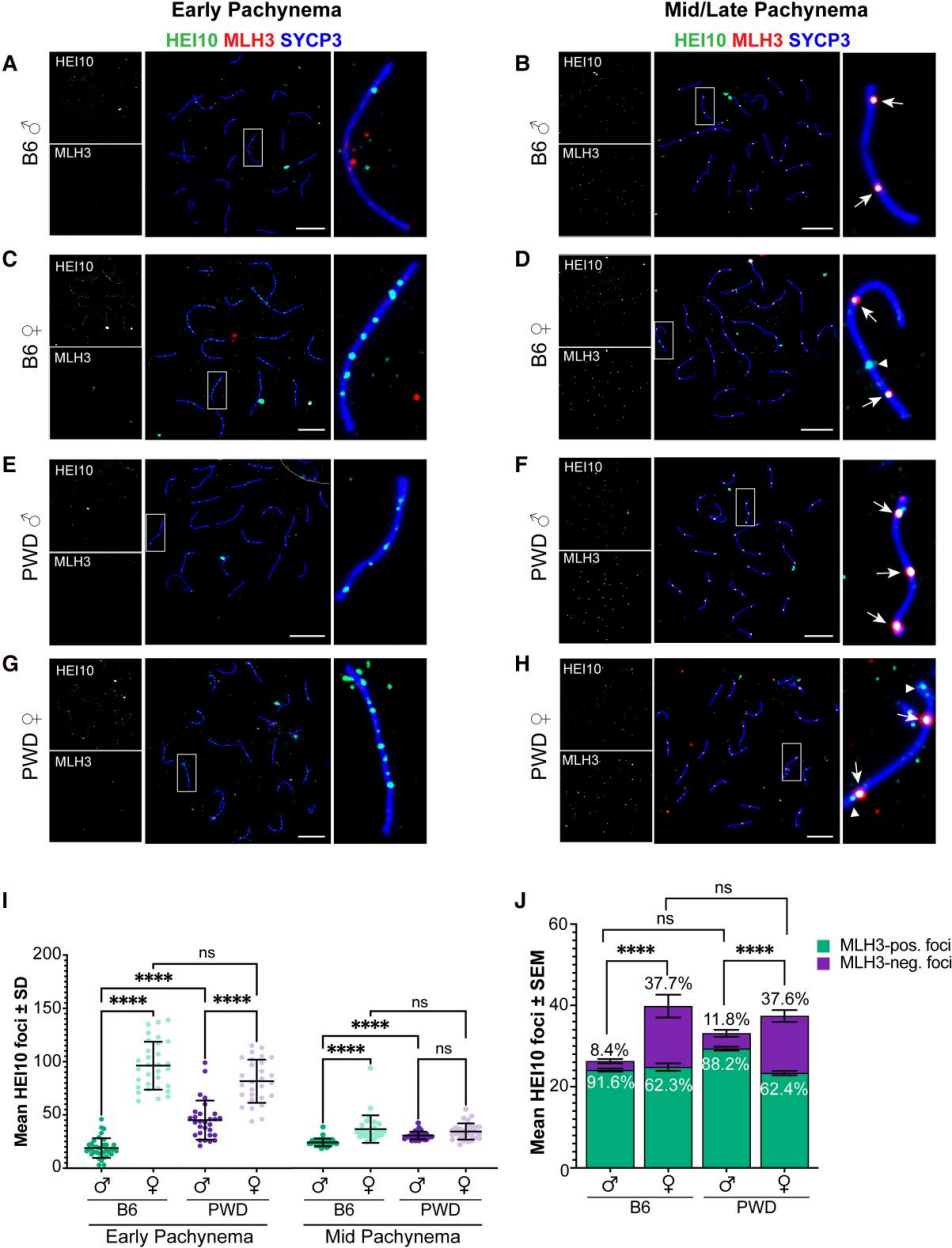
Sexual dimorphism in timing of HEI10 localization to the chromosome axis accumulation at presumptive class I crossover sites. Representative immunofluorescence images of early (a, c, e, g) and mid (b, d, f, h) pachytene spermatocytes and oocytes from B6 (a to d) and PWD (e to h) mice stained for HEI10 (green), MLH3 (red), and SYCP3 (blue). Individual HEI10 and MLH3 channels are shown in grayscale to the left of composite images; enlarged views of SCs within white boxes are shown at right. White arrows indicate HEI10 foci colocalized with MLH3; arrowheads indicate HEI10 foci without MLH3 colocalization. i) Significant differences in HEI10 foci number at early pachynema (*F* = 118.7, *P* < 0.0001) were observed between sexes in B6 (*t* = 17.88) and PWD (*t* = 7.12) mice; males also differed between strains (*t* = 7.00); samples sizes (animals [cells]): B6 male (3 [36]), female (3 [31]); PWD male (3 [29]), female (4 [28]). At mid-pachynema (*F* = 15.14, *P* < 0.0001), B6 males showed significantly fewer HEI10 foci than B6 females (*t* = 5.04) or PWD males (*t* = 7.17). j) The proportion of HEI10 foci colocalized with MLH3 at mid-pachynema showed significant sex differences (B6: χ^2^ = 234.8; PWD: χ^2^ = 188.0) but no strain differences); samples sizes (animals [cells]): B6 male (3 [35]), female (3 [29]); PWD male (3 [31]), female (3 [30]). Asterisks denote significant differences: ****P < 0.0001 (Games-Howell for i; Bonferroni for j). Error bars represent SD (i) or SEM (j). Scale bars represent 10 µm.

Finally, we wanted to identify a molecular explanation for the apparent sex and strain differences in class I CO maturation. Previous studies have identified several RING-E3 ligases, including HEI10, RNF212, and RNF212B, as critical to the stabilization of CO intermediates and designating which intermediates become class I COs (Ward et al. 2007; Reynolds et al. 2013; Qiao et al. 2014; Condezo et al. 2024; Ito et al. 2025)—and these proteins have also been identified in QTL studies as contributing to inter-species differences in meiotic CO levels (Kong et al. 2008; Sandor et al. 2012; Ziolkowski et al. 2017; Johnston et al. 2020). We thus hypothesized that differential RING-E3 ligase localization dynamics underpin sex and strain differences in CO maturation. However, due to a lack of commercially available antibodies, we could only examine HEI10 localization by indirect IF (Fig. 6a to h).

In early pachynema, we observed unexpected sex and strain differences in the timing of HEI10 localization to the SC. Females of each strain showed high numbers of HEI10 foci, markedly more than their ultimate number of MLH1 foci but fewer than early MSH4 (mean ± SEM: 96.2 ± 4.0 and 81.6 ± 3.8 for B6 and PWD females, respectively; Fig. 6i; Table 3). PWD males also exhibited a higher early HEI10 foci count than their eventual MLH1 foci count (45.1 ± 3.4), but significantly lower by comparison to PWD females (*P* < 0.0001; Fig. 6i; Table 3). In contrast, B6 males had fewer HEI10 foci in early pachynema than their eventual mean MLH1 count (18.9 ± 1.5) and significantly fewer than either B6 females (*P* < 0.0001) or PWD males (*P* < 0.0001; Fig. 6i; Table 3). These trends were consistent whether analyzing total HEI10 foci per nucleus or weighting them by SC length (Figure S6a; Table 3).

In mid-pachynema, HEI10 foci number increased in B6 males but decreased in all other groups. Previous studies have reported mid-pachytene spermatocyte HEI10 foci occur at near-identical levels as MLH1 in mice (Qiao et al. 2014; Condezo et al. 2024; Ito et al. 2025), and the males in our study were consistent with this. As such, significant differences in HEI10 foci number in mid-pachynema corresponded with differences in class I COs (Fig. 6i; Table 3). B6 males (26.3 ± 0.7) had significantly fewer HEI10 foci by comparison to B6 females (39.8 ± 2.6; *P* < 0.001) and PWD males (33.3 ± 0.7, *P* < 0.0001; Fig. 6i; Table 3). In contrast, B6 and PWD females (37.4 ± 1.5) did not significantly differ, and unexpectedly neither did PWD males and females (Fig. 6i; Table 3). As with our early pachynema HEI10 analysis, trends were consistent when foci counts were weighted by SC length (Figure S6a). As females had more mid-pachynema HEI10 foci than COs, we examined the colocalization of HEI10 with a marker for class I COs, MLH3 (Fig. 6b, d, f, h, j). Males in both strains showed higher proportions of HEI10 colocalizing with MLH3 than did their female counterparts (B6 males vs. females: 91.6% vs. 62.3%, *P* < 0.0001; PWD males vs. females: 88.2% vs. 62.4%, *P* < 0.0001; Fig. 6j). Oocytes lack clearly defined fiduciary markers to reliably substage pachynema; thus, there was a possibility the cells we analyzed for HEI10-MLH3 colocalization were not mid-pachytene or they were still in the process of CO designation. To rule out these possibilities, we compared MLH3 foci counts to our previous MLH1 foci counts and found them to be nearly identical (Figure S6c), suggesting these cells had achieved the expected number of class I COs for their sex and strain. Thus, these findings indicate a relatively high number of residual HEI10 foci in oocytes that do not localize to sites of COs and lend support to our hypothesis of sex and strain differences in the regulation of CO designation.

## Discussion

Over the last 25 years, sexual dimorphism in SC length has been the leading explanation for heterochiasmy among mammals (Lynn et al. 2002; Tease and Hultén 2004; Gruhn et al. 2013; Wang et al. 2017). Our analysis of diverse mouse strains provides nuance to this long-standing dogma. We find that males from most backgrounds have shorter SC lengths and stronger CO interference than their corresponding females and that these factors reinforce the lower rates of class I COs in these males relative to their female siblings. However, male PWD mice flout this rule and have higher CO frequency than females despite stronger interference and shorter axis length. Further, our analysis of key CO factors (RAD51, MSH4, and HEI10) indicates that sex- and strain-specific differences in the efficiency of CO designation underlie the exceptional sexual dimorphisms seen in PWD mice. Finally, our present study is the first to analyze both MLH1 foci in pachynema and chiasmata in diakinesis/metaphase I to evaluate sex differences in class I/II CO dynamics and distribution in mice from diverse genetic backgrounds.

### Interference and the centromere effect: reinforcing the sexual dimorphic relationship between axis length and CO rate

In meiosis, each chromosome is organized into a linear chromatin loop-axis array; sex differences in chromatin loop size impact the physical length of the axis. Interference further restricts the number of COs along the length of the axis, with previous studies suggesting short chromosomes and the PAR of the XY require numerous short loops arranged along a longer SC to produce the high numbers of DSBs required to ensure formation of the obligate CO (Kauppi et al. 2011; Wang et al. 2017, 2019; Murakami et al. 2020). Recent findings in mouse models have implicated sexually dimorphic accumulation of SUMO1 as a critical regulator of axis length and chromatin loop size (Yun et al. 2026), consistent with prior observations that the density of loops per micron SC is evolutionarily conserved (Zickler and Kleckner 2015). Variation in the dynamic organization of the loop-axis throughout prophase I may modulate sex and strain differences in CO rates. Future studies examining loop-axis organization—both globally and local to DSB hotspots—may provide greater insight into the effect of axis length on CO interference. Although the mechanisms regulating CO interference remain poorly understood, it has been postulated that the designation of one CO at one location results in the propagation of an interference signal spreading in either direction away from the CO over a definite micron distance along the chromosome axis; thus, CO number is thus predicted to positively scale with SC length (Petkov et al. 2004; Zickler and Kleckner 2016; Capilla-Pérez et al. 2021; von Diezmann and Rog 2021).

We predicted that the number of MLH1 foci would be limited by two complementary phenomena: (i) the centromere effect suppressing pericentromeric COs (pushing foci toward the distal end of the chromosome) and (ii) CO interference causing wider spacing between adjacent MLH1 foci (pushing COs apart and toward the telomeres) (Pazhayam et al. 2021; Wang et al. 2021; White et al. 2025). Under this model, chromosomes with multiple MLH1 foci would require a sufficient axis length to accommodate both phenomena. Applying this framework to our data, we find that mean mouse SC length (7 to 10 µm) approximates the sum of the median inter-foci distance (5 to 6 µm) and pericentromeric suppression (2 to 3 µm), leaving little allowance for multiple MLH1 foci per chromosome. This likely explains why most mouse chromosomes across all strains and sexes—except PWD males—have only one MLH1 focus. Critically, we observed males across mouse strains exhibit stronger interference than their corresponding females. In context of their shorter chromosome axes, the greater inter-CO distance results in a lower likelihood of chromosomes having multiple MLH1 foci, thus greatly limiting CO frequency in males.

Pericentric suppression of crossing over is a second driving force of CO patterning. For single-MLH1 focus chromosomes, males show more distal CO placements than females across all strains, consistent previous reports (Froenicke et al. 2002; Lynn et al. 2005; de Boer et al. 2015; Gruhn et al. 2016; Brick et al. 2018; Sardell and Kirkpatrick 2020). For chromosomes with multiple MLH1 foci, however, sex differences in the centromere effect are absent in most strains. Notably, the centromere effect in PWD females was stronger than in either their corresponding males or females from other strains. Thus, the pronounced sex differences in CO positioning between PWD mice likely result from weaker interference combined with a stronger centromere effect in females. PWK/PhJ mice (close relatives of PWD/PhJ) possess larger minor satellite arrays compared to the other strains we analyzed (Arora et al. 2021, 2023), and these ∼120 bp repeats define the core centromere where CENP-A binds (Arora et al. 2021, 2023). Given strain relatedness, we predict PWD mice likely have enlarged minor satellite domains. Depending on sex and strain differences in the compaction of minor satellite domains along the chromosome axis, this could be a factor in the larger centromere effect in PWD females. However, why only PWD females (and not males) show enhanced centromere effects remains unclear. Studies in *Drosophila* suggest centromeric repeat dosage does not alter the centromere effect (Pazhayam et al. 2024), and thus, the larger PWD minor satellite domain is likely not the primary driver of the centromere effect. Rather, pro-CO factors mei-218 and rec and species-specific variants of the SC protein C(3)G have been shown to influence pericentric CO patterning in *Drosophila* (Hawley et al. 2025; Hughes et al. 2025; Pazhayam et al. 2025). Similarly, the cohesin subunit Psc3 in fission yeast and pericentromeric cohesin enrichment directed by phosphorylation of the Ctf19 kinetochore complex in budding yeast have been shown to suppresses crossing over (Vincenten et al. 2015; Hinshaw et al. 2017; Nambiar and Smith 2018). Future investigation of sex-specific differences in pericentromeric chromatin architecture and cohesin distribution could reveal the molecular basis for sex and strain differences in the mouse centromere effect.

### What makes PWD mice so exceptional?

For most mouse strains, the data presented herein support the idea that CO interference and the centromere effect reinforce sex differences in SC length to drive heterochiasmy (Lynn et al. 2002; Kleckner et al. 2003; Tease and Hultén 2004; Gruhn et al. 2013; Ruiz-Herrera et al. 2017; Wang et al. 2017, 2019). However, data presented here and elsewhere (Dumont and Payseur 2011a; Peterson and Payseur 2021) show that PWD mice clearly flout this dogma, with males exhibiting much higher CO number than females despite having shorter axes and stronger CO interference. Our regression modeling reveals an outsized and inverted effect of sex on MLH1 foci number specifically in PWD mice (i.e. males show large positive association with MLH1 number, whereas most other strains show smaller negative relationship). Moreover, sex and SC length have nearly independent effects on MLH1 focus numbers in PWD mice, demonstrating that factors beyond SC length sexual dimorphism determine heterochiasmy in this strain. Notably, PWD mice are not alone in exhibiting higher male than female CO rates, as similar trends have been observed in SARB/NachJ, PWK/PhJ, GOR/TUA, and MSM/MsJ mouse strains (Peterson and Payseur 2021; Wooldridge et al. 2026). For future investigations, using these strains may provide critical insight into the evolution of the complex relationship between sexually dimorphic CO rates and chromosome axis lengths we have reported here.

Recent comparative analyses have indicated that recombination landscapes are rapidly evolving in mouse subspecies and populations, with *Prdm9* allelic variations driving divergence of DSB hotspots across groups (Wooldridge and Dumont 2023). At the level of CO rates, this divergence is strongly sex biased. Female CO rates are relatively conserved across strains; however, *M. m. musculus* males show signs of adaptive increases in CO rates by comparison to other subspecies (Wooldridge et al. 2026). Notably, the two primary genetic loci driving PWD × B6 F_1_ male, *Prdm9* and *Hstx2*, have roles in modulating meiotic recombination and CO rates (Flachs et al. 2012; Lustyk et al. 2019; Fotopulosova et al. 2024; Jansa et al. 2025). Recently, the microRNA cluster, *Mir465*, was identified as the sterility factor of the *Hstx2* locus (Lustyk et al. 2019; Jansa et al. 2025). In addition to acting as a meiotic checkpoint factor, deletion of *Mir465* in B6 mice causes increased male CO rates (Jansa et al. 2025). Paradoxically, *M. m. musculus* subspecies were found to have expanded *Mir465* clusters; thus, *Mir465* may actually depress CO rates in PWD males (Jansa et al. 2025). That genetic modifiers of meiotic recombination, CO rate, and checkpoint signaling can affect the severity of hybrid sterility (and thus contribute to reproductive isolation) further suggests that the sexually dimorphic CO rates observed in PWD and other *M. m. musculus* strains likely reflect broader subspecies-level differences in evolutionary regulation of meiotic recombination.

Using RAD51:MSH4 ratio as a proxy for CO licensing efficiency, we identified a sex difference in PWD but not B6 mice, indicating slightly higher licensing efficiency in PWD females. However, this difference only offset the initial sex difference in presumptive DSB induction. Rather, MSH4:MLH1 ratio analysis indicated higher CO designation is the primary driver of higher CO frequencies in PWD males vs. females. Our data show sex differences in HIE10 localization dynamics in B6 mice consistent with recent reports (Ito et al. 2025), while PWD males exhibit higher numbers of early HEI10 foci more similar to females. However, early HEI10 foci numbers do not correlate with strain-specific heterochiasmy patterns, as B6 and PWD females show similar HEI10 levels that exceed those seen in PWD males. Curiously, females from both strains show higher proportions of HEI10 foci in mid-pachynema that do not colocalize with class I COs (Fig. 6d, h, j), which may reflect sexually dimorphic CO designation efficiency.

Our observations of CO dynamics in PWD mice reveal that chromosome axis length is neither a universal determinant of CO frequency nor the only driver of heterochiasmy. HEI10 represents just one component in a complex regulatory network that varies by sex and genetic background and which might participate in determining CO frequency and distribution along with many other key pro-CO factors that have been shown in B6 mice to exhibit sex differences in their regulation during prophase I (e.g. CNTD1, RNF212, and RNF212B) (Reynolds et al. 2013; Condezo et al. 2024; Ito et al. 2025; Wood et al. 2025). Further, some pathogenic alleles of *Rnf212b* are linked to male and female infertility in humans, likely the result of critical disruption of the class I CO pathway (Gershoni et al. 2023; Kappy et al. 2024; Darko et al. 2025). Sequence variants in *Rnf212* and *Rnf212b* have also been associated with CO rate differences in humans and other mammals, with some polymorphisms having opposite effects on male and female CO frequency (Kong et al. 2008; Chowdhury et al. 2009; Johnston et al. 2018, 2020). It is possible, therefore, that allelic variants in these CO designation genes affect CO rates among diverse mouse strains in sex-specific manners. Future studies can use the genetic diversity of mouse strains to identify structure-function relationships among variants of these CO designation factors. The lack of a causal relationship between chromosome axis length and heterochiasmy in PWD mice highlights this strain as a critical model to further unravel the puzzle of sexual dimorphism in meiotic chromosome behavior. Analogous studies in other therian species may reveal that the relative contributions of subsets of these CO designation factors are adjusted in a species- and sex-specific manner to achieve optimal CO levels and distributions required for meiotic fidelity, as alluded to previously (Gray and Cohen 2016).

### Sexually dimorphic balance between CO classes: implications for CO homeostasis

Despite the very low rate of class II CO selection, this pathway appears to have a critical function in CO assurance as we observed unpaired univalents in diakinesis far less frequently than would be predicted from MLH1 focus counts through pachynema. This effect is particularly apparent in male mice of each strain. For instance, despite 12% to 16% of pachytene spermatocytes in DBA and CAST having at least one SC without an MLH1 focus, univalents were never observed among the diakinesis cells of these males (Figure S2c). The most logical explanation is that these SCs acquired class II Cos; otherwise, they would present as univalents. Rather than a universal sexual dimorphism in CO class usage rates, our data suggest that for a given strain, the sex with fewer class I COs tends to have more class II COs. It is critical to note, however, that because no reliable marker for class II COs is currently available, our findings represent an indirect assessment, and the differences in quantification methods between MLH1 and chiasmata limit the precision with which class II COs can be quantified. Thus, the interpretations presented herein should be understood within this methodological context. Nevertheless, our findings are suggestive that the two CO pathways are not independent and are likely regulated to ensure a balanced CO homeostasis. Previous studies using *Mus81-*null mice support this notion, finding that loss of class II COs elicits an upregulation of the class I pathway to maintain normal chiasmata number (Holloway et al. 2008). Our data also demonstrate that mice with higher class I rates—which also tend to have the longer SC lengths—have lower rates of class II COs. Because class II COs are thought to be interference independent (Munz 1994; Cromie et al. 2006; Holloway et al. 2008; Gray and Cohen 2016), they are likely more restricted by the number of class I COs than by the axis length. This idea fits with recent work from our lab showing mice with defective MSH5 ATP binding lose all COs in diakinesis, suggesting that CO homeostasis likely involves the combination of both CO classes, with MutSγ serving as a hub for pathway selection (Milano et al. 2019). Importantly, the high rate of MutSγ deposition in zygonema/early pachynema relative to the final MutLγ focus count in pachynema in mammals (Kneitz et al. 2000; Cole et al. 2012; Milano et al. 2019) lends support to this conclusion, indicating that the selection of final CO sites, regardless of their classification, occurs from a supernumerary population of MutSγ-defined CO intermediates.

Taken together, these findings show that sexual dimorphism in crossing over results from the combined effects of structural and regulatory processes that vary across genetic backgrounds, indicating that no single mechanism can fully explain the diversity of CO outcomes in mice. Understanding how these mechanisms are coordinated in different sexes and genetic contexts will be critical for determining the evolutionary pressures that drive heterochiasmy and homeostasis between CO pathways.

## Materials and methods

### Care and use of experimental animals

Breeding stocks of adult inbred C57BL/6J (000664), DBA/2J (000671), 129S1/SvImJ (002448), CAST/EiJ (000928), and PWD/PhJ (004660) mice were procured from The Jackson Laboratory. For each mouse strain, brother/sister breeding pairs were mated beginning at 6 weeks of age. Resulting offspring were weaned at 3 weeks of age, separated by sex, and housed in polysulfone cages (Allentown PC75JHT) on ventilated racks. No more than five mice were housed per cage. Each cage contained Bed-o’-cobs ¼″ bedding (Scotts 330BB), Bio-Serv standard hut (Scotts K3352), and Crinkle Nest Kraft (Scotts CNK), and three 1″×1″ nestlets (Scotts NES7200) for enrichment. All mice were maintained in a climate-controlled environment with a 12 h light/dark cycle and provided food (Envigo Bioproducts Inc. Rodent diet 7012) and water ad libitum. All adult and juvenile mice were euthanized using inhaled CO_2_ for at least 3 min and monitored for cessation of breathing, followed by secondary internal cervical dislocation. All adult males in this study were euthanized between 10 and 15 weeks of age. Fetal mice were euthanized by decapitation. Juvenile female mice used for oocyte chiasmata analysis were euthanized between post-natal day (PND) 24 and 28. All fetal mice in this study were euthanized between 16.5- and 18.5-d post coitum (dpc) by decapitation. Animal handling and procedures were performed following approval by the Cornell Institutional Animal Care and Use Committee under protocol 2004–0063.

### Prophase I chromosome preparations

Meiotic chromosome preparations were made according to methods initially developed by Peters and colleagues and refined as described previously (Peters et al. 1997; Horan et al. 2024; Horan 2025). Testes were cleaned and detunicated in PBS. Seminiferous tubules were teased apart and incubated in hypotonic extraction buffer, pH 8.2 to 8.4 (30 mM Tris-HCl; 50 mM sucrose; 17 mM trisodium citrate dihydrate; and 5 mM EDTA) for 20 min on ice. For every two slides made, approximately 7 mm of tubule was macerated in 60 µL of 500 mM sucrose at room temperature. Slides were coated in a thin layer paraformaldehyde (1% PFA, 0.15% Triton X, pH 9.2 to 9.3), and 20 µL of cell suspension was spread on each slide. Fetal ovaries were cleaned in PBS and incubated in hypotonic extraction buffer for 12 to 15 min at room temperature. Both ovaries were macerated together in 35 µL of 500 mM sucrose. Slides were divided into three equivalent-sized square wells using a hydrophobic PAP pen and dipped into 1% PFA (0.15% Triton X, pH 9.2 to 9.3). For each square, 5 µL of cell suspension was spread across the section. Slides were incubated in a room temperature humid chamber for 2 h before being removed from the humid chamber and left to completely air-dry. Dried slides were washed for 2 min in Milli-Q water with 0.4% Photo-flo 200 solution (Kodak Professional 1464510) and immediately immunostained.

### IF staining

Indirect IF staining was performed as described previously (Horan et al. 2024; Horan 2025). Slides were washed for 10 min in PBS with 0.4% Photo-flo, then 10 min in PBS with 0.1% Triton X, and finally blocked for 10 min in 10% antibody dilution buffer (ADB; 100% stock consisted of 3% (w/v) bovine serum albumin [Millipore Sigma A5177-5EA], 10% (v/v) normal goat serum [Gibco 16210-072], 0.05% Triton X in PBS and sterilized using a 45 µm syringe filter). Primary antibodies were diluted in 100% ADB, applied to slides, and incubated in a room temperature humid chamber overnight. Slides were washed as before. Secondary antibodies were diluted and applied in a similar manner as the primary antibodies and incubated in a room temperature humid chamber for 2 h in the dark. Slides were washed 3 × 5 min in PBS with 0.4% Photo-flo, 1 × 5 min in Milli-Q water with 0.4% Photo-flo and mounted using ProLong Diamond antifade with DAPI (Invitrogen P36962). Commercially available antibodies and dilutions used in this study are listed in Table S6, along with two custom antibodies previously made and used by the Cohen lab at Cornell University: guinea pig anti-MLH3 (Horan et al. 2024) and rabbit anti-SYCP3 (Kolas et al. 2005).

### Chromosome preparations from diakinesis spermatocytes

Chromosome preparations from diakinesis spermatocytes were made according to methods developed by Evans and colleagues (Evans et al. 1964) and as detailed previously (Horan et al. 2024). Seminiferous tubules were placed in 2.2% sodium citrate and minced using scalpel blades for approximately 30 s. Tubules were transferred to a 15 mL conical tube and further dissociated by repeated pipetting. Samples were left to settle at room temperature for 15 min, and the supernatant was collected and centrifuged at 600 ´ *g* for 5 min at room temperature. The cell pellet was resuspended in 4 mL of 1.1% sodium citrate and incubated at room temperature for 15 min. The sample was centrifuged as before, the supernatant discarded, and the pellet was vortexed into suspension as a fixative (75% methanol, 25% glacial acetic acid) was added dropwise. After two additional rounds of centrifugation and resuspension in the fixative, cells were dropped from approximately 2 m onto slides and air dried.

Oocyte diakinesis/prometaphase I chiasmata spreads were done as detailed previously (Horan 2025). Dictyate-arrested oocytes were isolated from the ovaries of unstimulated juvenile females. Ovaries were dissected into warmed, pre-gassed EmbryoMax M2 Medium (Millipore-Sigma MR-015) containing 2.5 µM milrinone (Millipore-Sigma M4659) to prevent meiotic resumption. Germinal vesicle (GV) oocytes were isolated using a 100-µm-wide Stripper Tip (Cooper Surgical MXL3-100) and collected in 20 µL drops of M2 medium with milrinone under EmbryoMax Filtered Light Mineral Oil (Millipore-Sigma ES-005). To induce meiotic resumption, denuded GV oocytes were washed by five serial passages through EmbryoMax Potassium Simplex Optimized Medium (KSOM; Millipore-Sigma MR-101-D). Washed GV oocytes were incubated in 20 µL KSOM drops under mineral oil (maximum 30 oocytes per 20 µL medium) at 37 °C in 5% CO_2_ for 5 h to reach diakinesis/prometaphase I. Oocytes were incubated in 0.9% sodium citrate hypotonic solution for 5 to 10 min before being transferred to a 1 µL drop of acidified water (eight drops glacial acetic acid in 50 mL ultrapure H_2_O) on a microscope slide. Excess liquid surrounding the oocyte was removed, and oocytes were burst by dropping fixative (75% methanol, 25% glacial acetic acid) from a 150 µm Stripper Tip (Cooper Surgical MXL3-200) and air dried. All chiasmata spreads were stained with 4% Giemsa (Millipore-Sigma GS500), washed 3 × 3 min in ddH_2_O, dried, and mounted with permount (Fisher Scientific SP15-100).

### Image acquisition

All slides were imaged using a Zeiss Axio Imager.Z2 with Colibri 7 epifluorescence microscope at 63× magnification. For IF experiments, DAPI (385 nm), AF488 (475 nm), RR-X (555 nm), and AF647 (630 nm) were imaged sequentially using standardized exposure times for each antibody. Images were processed and adjusted using Zeiss Zen Blue version 3.0 (Carl Zeiss AG, Oberkochen, Germany) to standardize brightness and background across all images. SYCP3 and MLH3 were used to stage prophase I cells. In spermatocytes, the extent of X and Y chromosome synapsis was used to substage pachynema into early, mid, and late, as described in previous work (Quack and Noel 1977; Turner et al. 2004). Pachytene oocytes from 17.5 dpc were considered early if all chromosome pairs were fully synapsed and MLH3 foci were present on 10 or fewer SCs. Slides stained with Giemsa were imaged using brightfield and processed using Zeiss Zen Blue version 3.0 (Carl Zeiss AG, Oberkochen, Germany).

### SC measurement, foci quantification, and statistical analysis

For each chromosome pair (excluding the XY in spermatocytes), chromosome axis lengths were measured using Zeiss Zen Blue version 3.0 (Carl Zeiss AG, Oberkochen, Germany) by manually tracing SYCP3 beginning at the centromere (marked with CREST or DAPI-dense spots). For each cell, genome-wide SC length was calculated by summing lengths for all 20 chromosomes (for females) or all 19 autosomes (males). At least 20 cells were measured per animal and at least three animals per group (Table 2; Table S1). Group differences in mean SC length were analyzed by Brown-Forsythe one-way ANOVA to account for unequal variance among sexes and strains; statistically significant differences (*P* < 0.05) between groups were determined by Games-Howell's multiple comparison test.

For MLH1, MSH4, HEI10, and RAD51, at least two independent observers who were blinded to the mouse strain manually counted foci using Photoshop (Adobe). Any focus that was at least 50% localized to SYCP3 was counted. MLH1, MLH3, MSH4, and HEI10 were additionally quantified by manually counting the fluorescence peaks for each antibody observed along tracings of chromosome axes (SYCP3). A flexible, manual thresholding of fluorescence intensity was used to define peaks as (i) being clearly defined (at least 50% brighter than background signal) and (ii) corresponding to visible foci as determined by manual comparison between images and fluorescence profile. Minor differences between foci and peak counts were reconciled on a per-focus basis, and cells with major scoring discrepancies, poor staining, or synaptic defects were excluded from analysis.

For MLH1, the distance from the centromere to each fluorescence peak was recorded for all SCs except the XY in spermatocytes. MLH1 foci were counted from 20 to 30 mid-pachytene cells per animal and at least three animals per group (Tables 1 and 2; Tables S1 to S4). Group differences in mean MLH1 foci counts were analyzed by Brown-Forsythe one-way ANOVA to account for unequal variance among sexes and strains; statistically significant differences (*P* < 0.05) between groups were determined by Games-Howell's multiple comparison test. Group differences in the frequency of cells containing an SC without an MLH1 focus (E0s) was done by χ² test (10 × 2 contingency table) for independence. Correlation between SC length and MLH1 foci number was done for each sex and strain using simple linear regression. To assess the relationships between sex and SC length on MLH1 foci number, a multiple regression analysis was done for each mouse strain. For each regression analysis, significance of the model (*P* < 0.05) was determined by F-test. To assess the distribution of MLH1 foci, SCs were grouped by whether they had 0, 1, or >1 foci. The proportion of SCs in each group were further categorized by length into “short,” “medium,” and “long” classes based on within-cell quartiles of total SC length (i.e. the longest and shortest five SCs and the intermediate-length 9 to 10 SCs). The relationship between SC length and MLH1 foci number were assessed by logistic regression performed in R (v4.5.1). Models were fitted using a binomial link function to assess the effects of sex, strain, and SC length (continuous or categorical) on the likelihood of multiple MLH1 foci per SC. Interaction terms were evaluated by likelihood ratio tests, and estimated marginal means were used to compare predicted probabilities among groups.

MSH4 and HEI10 foci in early and mid-pachynema were analyzed only for C57BL/6J and PWD/PhJ males and females. Colocalization of MSH4-MLH3 and HEI10-MLH3 in early and mid-pachynema was done by quantifying MSH4- or HEI10-exclusive, MLH3-exclusive, and colocalized foci/peaks. Foci were counted from 8 to 15 cells per substage per animal, and at least three animals per group (Table 3). Statistical analysis of both total foci counts and counts normalized by SC length were done for MSH4 and HEI10. For MSH4 data from each substage (exhibiting normal distribution), group differences in mean foci counts were analyzed by Brown-Forsythe one-way ANOVA to account for unequal variation among sexes and strains; statistically significant differences (*P* < 0.05) between groups were determined by Dunnett's T3 multiple comparison test to accommodate the small sample size (*n* < 50 cells). For HEI10 data (not normally distributed), group differences in median focus counts were analyzed by Kruskal-Wallis test; statistically significant differences (*P* < 0.05) between groups were determined by Dunn's multiple comparison test. Colocalization of MHS4-MLH3 and HEI10-MLH3 was evaluated by χ² tests of independence applied to 2 × 2 contingency tables as expected counts exceeded recommended thresholds. Comparisons were performed between sexes within a strain and between strains within each sex, and *P*-values were adjusted for multiple comparisons using Bonferroni and false discovery rate (FDR) corrections.

Analysis of RAD51 foci was done only for C57BL/6J and PWD/PhJ males and females. RAD51 foci were quantified in zygotene cells with less than 50% synapsis. At least three animals were analyzed per group, with 15 to 20 cells per animal (Table 3). Group differences in mean foci counts were analyzed by Brown-Forsythe one-way ANOVA to account for unequal variation among sexes and strains; statistically significant differences (*P* < 0.05) between groups were determined by Games-Howell's multiple comparison test.

Differences in the relative abundances for each marker pair (RAD51 vs. MLH1, MSH4 vs. MLH1, and RAD51 vs. MSH4) were performed using generalized linear mixed models (GLMMs). For each marker pair, foci counts were modeled assuming a negative binomial distribution with a log link. The model included marker identity, sex, strain, and their interactions as fixed effects and mouse identity as a random intercept to account for repeated observations from the same animal:


$$Y_{ijkl}∼NegBin(\mu_{ijkl},\;\theta )$$

$$\begin{array}{rl} \log\;(\mu_{ijkl})\;= & \beta_{0}+\beta_{1}Marker_{j}+\beta_{2}Sex_{k}+\beta_{3}Strain_{m}\\ & +\;\beta_{4}(Marker_{j}\times Sex_{k})+\beta_{5}(Marker_{j}\times Strain_{m})\\ & +\;\beta_{6}(Sex_{k}\times Strain_{m})+\beta_{7}(Marker_{j}\times Sex_{k}\times Strain_{m})+b_{i} \end{array}$$

where *Y_ijkl_* represents the observed number of foci and *b_i_∼N(0, σ^2^)* represents the random effect of mouse. Estimated marginal means were obtained for each Marker × Sex × Strain combination using the emmeans package in R (v4.5.1). Pairwise comparisons of ratios between markers were assessed on the log scale with standard errors (SEs) calculated using the delta method. Significant differences among group log-ratios were determined using Wald tests with Bonferroni and FDR corrections for multiple comparisons.

### Quantification of chiasmata, calculation of class II CO number, and statistical analysis

Quantification of chiasmata from all chromosome pairs (excluding the XY in spermatocytes) was done according to the previously published rubric (Horan 2025). Due to the limited yield of oocytes from juvenile ovaries, chiasmata were assessed from a minimum of 25 oocytes pooled from at least three individuals. For males, chiasmata were assessed from at least three individuals, 10 to 15 spermatocytes per individual. Group differences in mean chiasmata counts were analyzed by Brown-Forsythe one-way ANOVA to account for unequal variance among sexes and strains; statistically significant differences (*P* < 0.05) between groups were determined by Games-Howell's multiple comparison test (Table 1; Table S1). For each sex and strain, the number of class II COs was inferred by subtracting the mean MLH1 foci number from the mean chiasmata number for each group. The SE was calculated by error propagation assuming independence between measures. Within each strain, male vs. female differences in calculated class II CO frequency were done by *z*-test based on the difference of means and pooled SE.

### Analysis of interference length, inter-MLH1 focus distances, and MLH1 focus to centromere length

For all SCs measured, the distance from the centromere to each MLH1 focus was recorded. An initial assessment of CO interference was done by calculating the distances between MLH1 foci for SCs with more than one MLH1 focus. The cumulative fraction of inter-focus distances was measured both as a percentage of SC length and in microns. Differences in the probability distributions of inter-focus distances between strains and sexes were determined by KS test, and statistically significant differences (*P* < 0.05) between groups were determined by Bonferroni post hoc test (Table S4). To determine if inter-focus distance differed for long and short chromosomes, we repeated the above analyses using the five longest and the five shortest SCs per cell. To assess pericentromeric suppression of crossing over, this same statistical analysis was done using distance from the centromere to the first MLH1 focus—both for SCs with only one focus and for SCs with more than one focus (Table S5).

For SCs with two MLH1 foci, focus position distributions along the SC were analyzed in R (v4.5.1). For each SC, positions of individual MLH1 foci were normalized to SC length (0.0 to 1.0, beginning at the centromere), and the normalized positions were plotted as density-scaled histograms by sex and strain. To determine the relative effects of sex (female as the reference group) and SC length on MLH1 foci count, we performed independent multivariable linear regression analyses for each strain. Each focus was classified as “terminal” (≤0.25 or ≥0.75) or “interior” (0.25 to 0.75), and differences in MLH1 focus position were assessed using contingency table analysis. For comparisons between sexes within each strain, where some expected counts were small, data were analyzed using Fisher's exact tests. Global differences in MLH1 focus position among strains within each sex were evaluated using χ² tests of independence, with pairwise strain comparisons using Fisher's exact tests on 2 × 2 contingency tables to provide exact *P*-values. Resulting *P*-values were adjusted using Bonferroni correction for multiple comparisons.

Overall CO interference strength and the evenness of MLH1 spacing was calculated using R (v4.5.1) by fitting inter-focus distances to the gamma distribution and comparing shape parameter (*v*). Distances between MLH1 foci were calculated as


$$d_{i}=x_{i+1}-x_{i}$$

where *x_i_* and *x_i_*  *_+_*  *_1_* represent positions of consecutive foci along the chromosome axis. Because the terminal interval between the last focus and the chromosome end does not contain a subsequent MLH1 focus, this final segment represents an incomplete waiting distance and was thus treated as a right-censored observation in the likelihood model:


$$c=L-x_{n}$$

where *L* is the SC length and *x_n_* is the position of the final MLH1 focus. Each SC, therefore, contributed one right-censored interval (the distance from the last or only MLH1 focus to the distal end of the SC), while SCs with multiple MLH1 foci also contributed uncensored intervals (the distances between adjacent foci). Interval distances were normalized to SC length, and their distribution was fit to a gamma distribution:


$$f(d;v,\theta )=\frac{d^{v-1}e^{\frac{-d}{\theta }}}{\Gamma (k)\theta^{v}}$$

where *v* is the shape parameter (with *v* = 1 corresponding to no interference) and *θ* is the scale parameter. Model parameters were estimated by maximum likelihood. Uncensored intervals contributed the gamma density *f(d_i_;k, θ)* to the likelihood while right-censored intervals contributed the survival probability:


S(c;k,θ)=1−F(c;k,θ)

where *F* is the cumulative gamma distribution. The total log-likelihood was


$$\log\;L=\underset{i}{\sum }\;\log\;f(d_{i};k,\theta )+\underset{j}{\sum }\;\log\;S(c_{j};k,\theta )$$

Initial parameter estimates were obtained by method-of-moments and optimized using Broyden–Fletcher–Goldfarb–Shanno (BFGS) minimization of the negative log-likelihood. Within each strain, likelihood ratio tests compared sex-specific shape parameters (distinct *v*, shared *θ*) to a pooled model with both parameters shared between sexes. Significance was assessed from the Χ^2^ distribution.

## Supplementary Material

msag130_Supplementary_Data

## Data Availability

The data underlying this article are available in the article and in its online Supplementary material. Raw data and R scripts will be shared on reasonable request to the corresponding author.
