# Supplementary material for: Molecular assessment of recombination processing across genetically diverse mouse strains reveals sexually dimorphic determinants of crossover distribution beyond chromosome length
Source: Mol Biol Evol. 2026 Jun 2;43(6):msag130. doi: 10.1093/molbev/msag130 (PMC13267643; doi:10.1093/molbev/msag130)
Supplement: msag130_Supplementary_Data [file msag130_supplementary_data.zip › Supporting Information.pdf]

Supporting Information

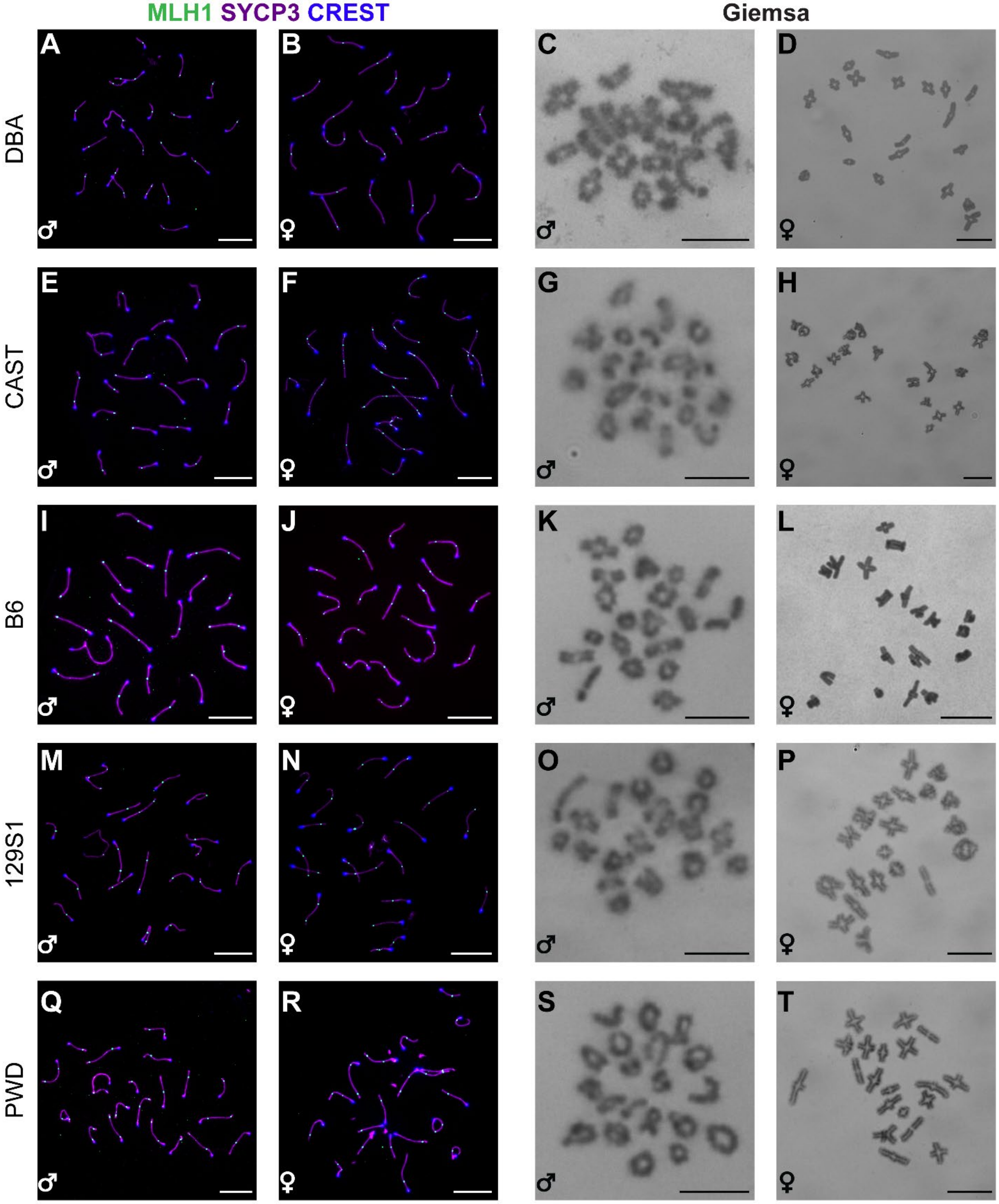

Fig. S1 Imaging of MLH1 and chiasmata Representative images of spermatocytes and oocytes for DBA (A-D), CAST (E-H), B6 (I-L), 129S1 (M-P), and PWD (Q-T) mice. (A-B, E-F, I-J,

M-N, and Q-R) pachytene chromosome spreads stained for MLH1 (class I COs, green), SYCP3 (chromosome axis, magenta), and CREST (centromeres, blue). (C-D, G-H, K-L, O-P, S-T) Giemsa-stained chiasmata spreads from diakinesis/metaphase I chromosome spreads. Scale bars represent 10 μm.

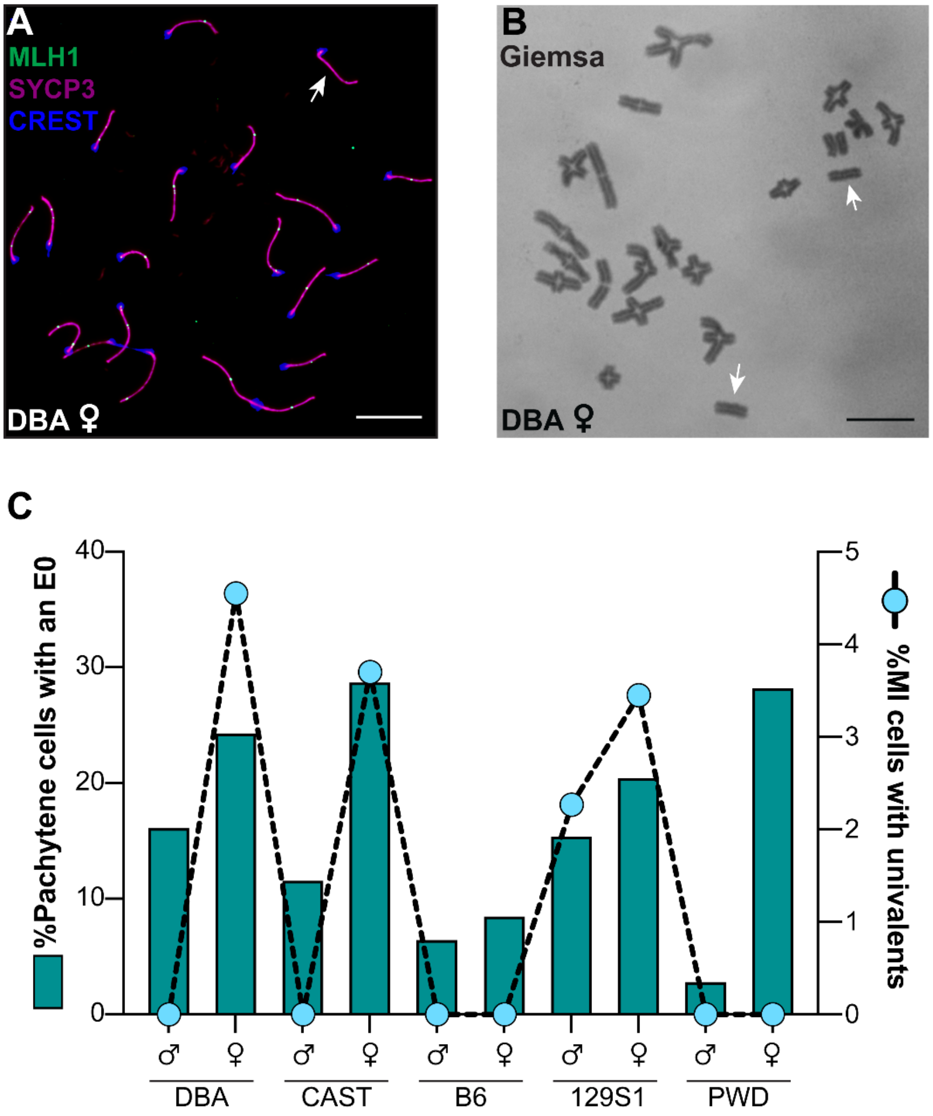

Fig. S2 The class II pathway facilitates assurance of the obligate crossover. (A) Representative immunofluorescence image of a DBA oocyte in which one chromosome pair failed to make a class I CO (E0, white arrow). (B) Representative Giemsa-stained DBA oocyte chiasmata spread showing unpaired homologous chromosomes (univalents, white arrow). (C) Percentage of pachytene cells with at least one E0 SC (dark teal bars, left axis) and percentage of metaphase I cells with univalents (light teal circles, right axis). Although chromosome pairs without a class I CO—predicted to result in unpaired chromosomes at metaphase I that likely mis-segregate—are relatively common, the actual incidence of metaphase I cells with univalents

is strikingly low across strains and sexes, indicating that in many meiotic cells, the obligate CO is made via the class II pathway. Scale bars, 10 μm.

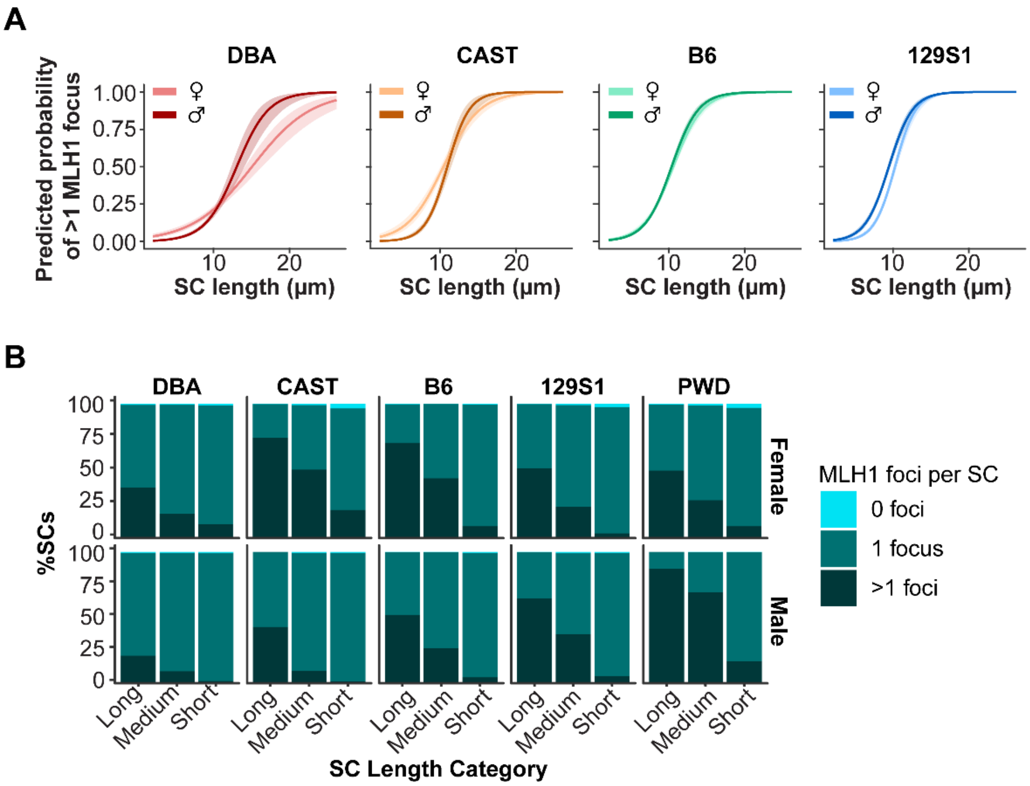

C

SC Length (μm)	Strain	Odds Ratio	SE	z-ratio	p-value
	DBA	3.589	0.93	4.96	<0.0001
	CAST	6.605	2.22	5.62	<0.0001
5 μm	B6	1.165	0.22	0.83	0.409
	129S1	0.381	0.09	-4.16	<0.0001
	PWD	0.362	0.07	-5.12	<0.0001
	DBA	1.184	0.14	1.47	0.142
	CAST	1.670	0.23	3.78	<0.001
10 μm	B6	0.912	0.07	-1.26	0.207
	129S1	0.583	0.06	-5.01	<0.0001
	PWD	0.129	0.01	-23.39	<0.0001
	DBA	0.391	0.12	-2.95	<0.01
	CAST	0.422	0.18	-2.07	<0.05
15 μm	B6	0.714	0.16	-1.54	0.124
	129S1	0.892	0.31	-0.33	0.745
	PWD	0.046	0.01	-11.88	<0.0001

Fig. S3 SC length affects sex differences in the likelihood of multiple crossovers per chromosome pair in most mouse strains. (A) Effect of SC length on the predicted probability of an SC having more than one MLH1 focus for DBA (red), CAST (orange), B6 (green), 129S1

(blue), and PWD (purple) males (dark) and females (light). Logistic regression analysis and significance are in Table S3. (B) Each SC per nucleus was classified as long (5 longest), short (5 shortest), or medium (9-10 intermediate-length). The percentage of SCs in each length category without an MLH1 focus (light teal), with 1 focus (teal), or with multiple foci (dark teal) is plotted for each sex and strain. (C) Logistic regression analysis of the effects of sex, strain, and SC length (continuous variable) on the likelihood of an SC having multiple MLH1 foci (B6 female as reference). This model predicted the likelihood of multiple MLH1 foci for SCs of three lengths: 5, 10, and 15 μm. Odds ratios below 1.0 indicate higher likelihood in males. Note that 15 μm SCs were exceptionally rare in males (absent in DBA males).

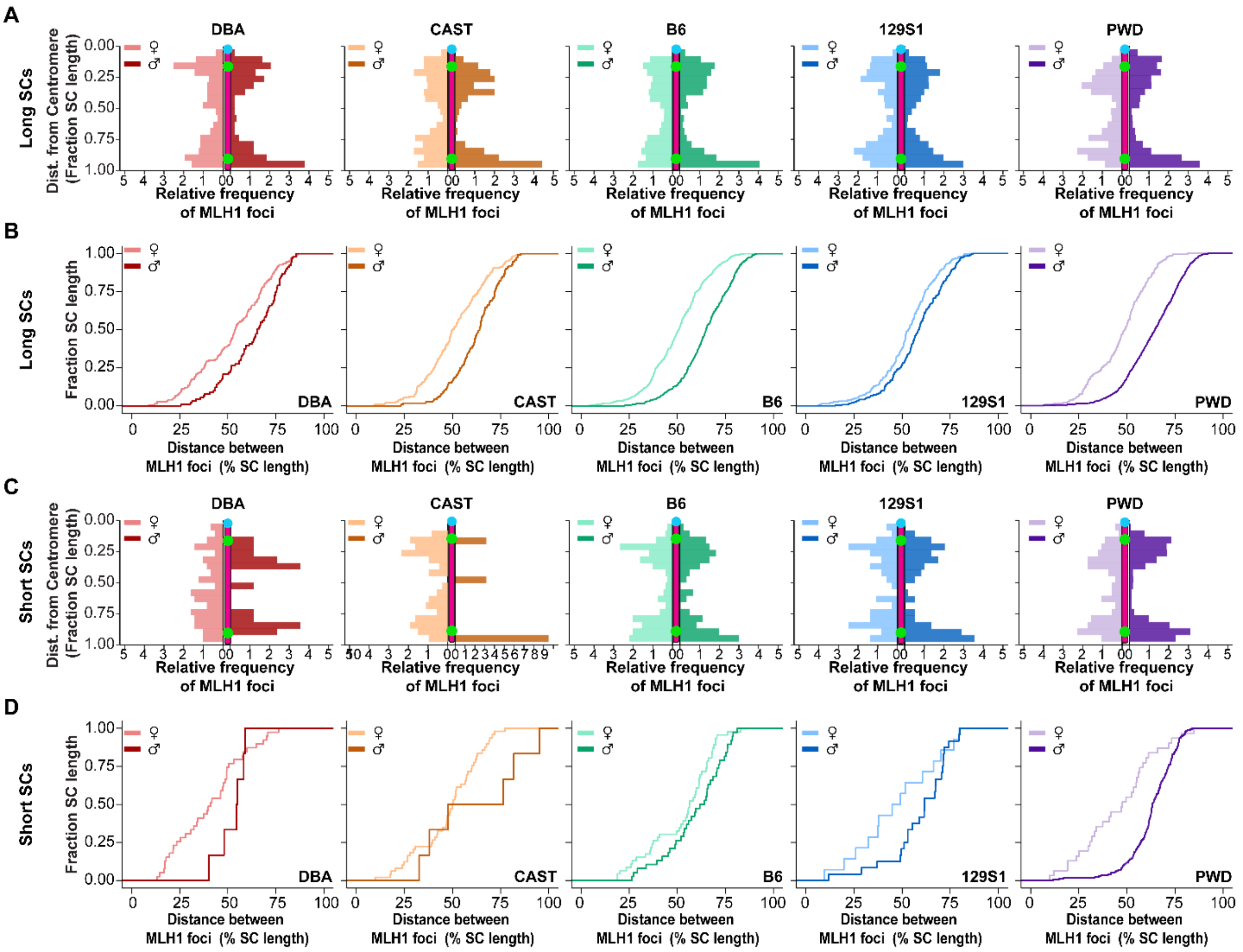

Fig. S4 CO interference is stronger in males from all strains for long SCs, but only in PWD males for short SCs. Expanded analysis from Fig. 3 examining CO interference on the longest (A-B) and shortest (C-D) five SCs per cell. Histograms (A, C) show the relative frequency of MLH1 foci at terminal SC ends (0.0–0.25 and 0.75–1.0). The y-axis represents fractional SC length from centromere (0.0) to distal telomere (1.0). (A) For long SCs, males (dark bars) had significantly more terminally placed MLH1 foci than females (light bars; Fisher's exact test): 74.43% vs. 65.15% in DBA (OR = 0.64, $p < 0.05$), 69.44% vs. 58.05% in CAST (OR = 0.61, $p < 0.05$), 72.80% vs. 59.05% in B6 (OR = 0.54, $p < 0.0001$), and 74.61% vs. 55.60% in PWD (OR = 0.35, $p < 0.0001$), but not in 129S1 (66.92% vs. 63.14%, $p = 0.3$). (C) Among short SCs, only PWD males showed greater terminal placement (75.72% vs. 55.56%; OR = 0.40, $p < 0.0001$). No significant sex differences were observed in other strains (male vs. female: DBA,

56.25% vs. 52.17%, $p = 0.79$; CAST, 83.33% vs. 61.59%, $p = 0.41$; B6, 68.42% vs. 66.19%, $p = 0.71$; 129S1, 69.64% vs. 64.29%, $p = 0.52$). (B) ECDF graphs of standardized inter-focus distance (%SC length) for long SCs show significantly greater distances in males for all strains (Kolmogorov-Smirnov: DBA, KS = 0.32, $p < 0.01$; CAST, KS = 0.35, $p < 0.0001$; B6, KS = 0.40, $p < 0.0001$; 129S1, KS = 0.21, $p < 0.01$; PWD, KS = 0.43, $p < 0.0001$). (D) For short SCs, only PWD males showed significantly greater inter-focus distance than females (KS = 0.49, $p < 0.001$).

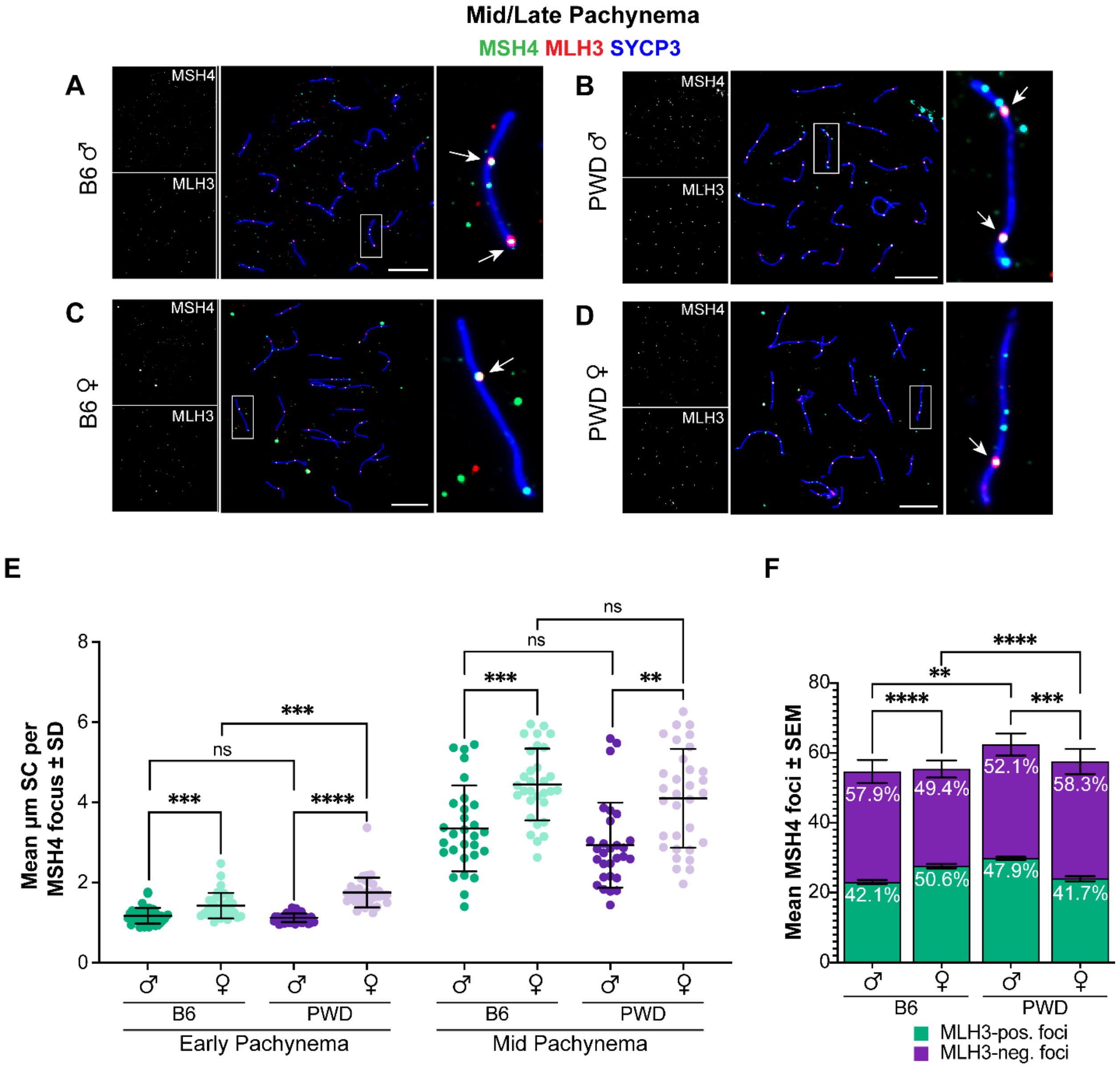

Fig. S5 Sex and strain differences in the density of MSH4 foci and their colocalization with MLH3 Representative images of mid-pachytene spermatocytes (A-B) and oocytes (C-D) from B6 (A, C) and PWD (B, D) mice stained for MSH4 (green), MLH3 (red), and SYCP3 (blue). (E) Comparison of mean microns of SC per MSH4 focus for B6 and PWD males and females at early ($F = 45.11$, $p<0.0001$) and mid-pachynema ($F = 12.46$, $p<0.0001$). Significant sex differences in mean microns/focus were observed in B6 (early: $t = 4.14$; mid: $t = 4.29$) and PWD (early: $t = 9.91$; mid: $t = 3.98$). Significant strain differences were only observed between

females at early pachynema ($t = 4.05$); samples sizes (animals [cells]): B6 male (3 [29]), female (3 [31]); PWD male (3 [30]), female (3 [32]). (F) The proportion of MSH4 foci colocalized with MLH3 at mid-pachynema showed significant sex differences (B6: $\chi^2 = 23.1$; PWD: $\chi^2 = 13.9$) and strain differences (males: $\chi^2 = 11.1$; females: $\chi^2 = 27.5$). Asterisks denote significant differences: **** $p < 0.0001$, *** $p < 0.001$, ** $p < 0.01$ (Games-Howell for E; Bonferroni for F). Error bars represent SD (E) or SEM (F). Scale bars represent 10 μm.

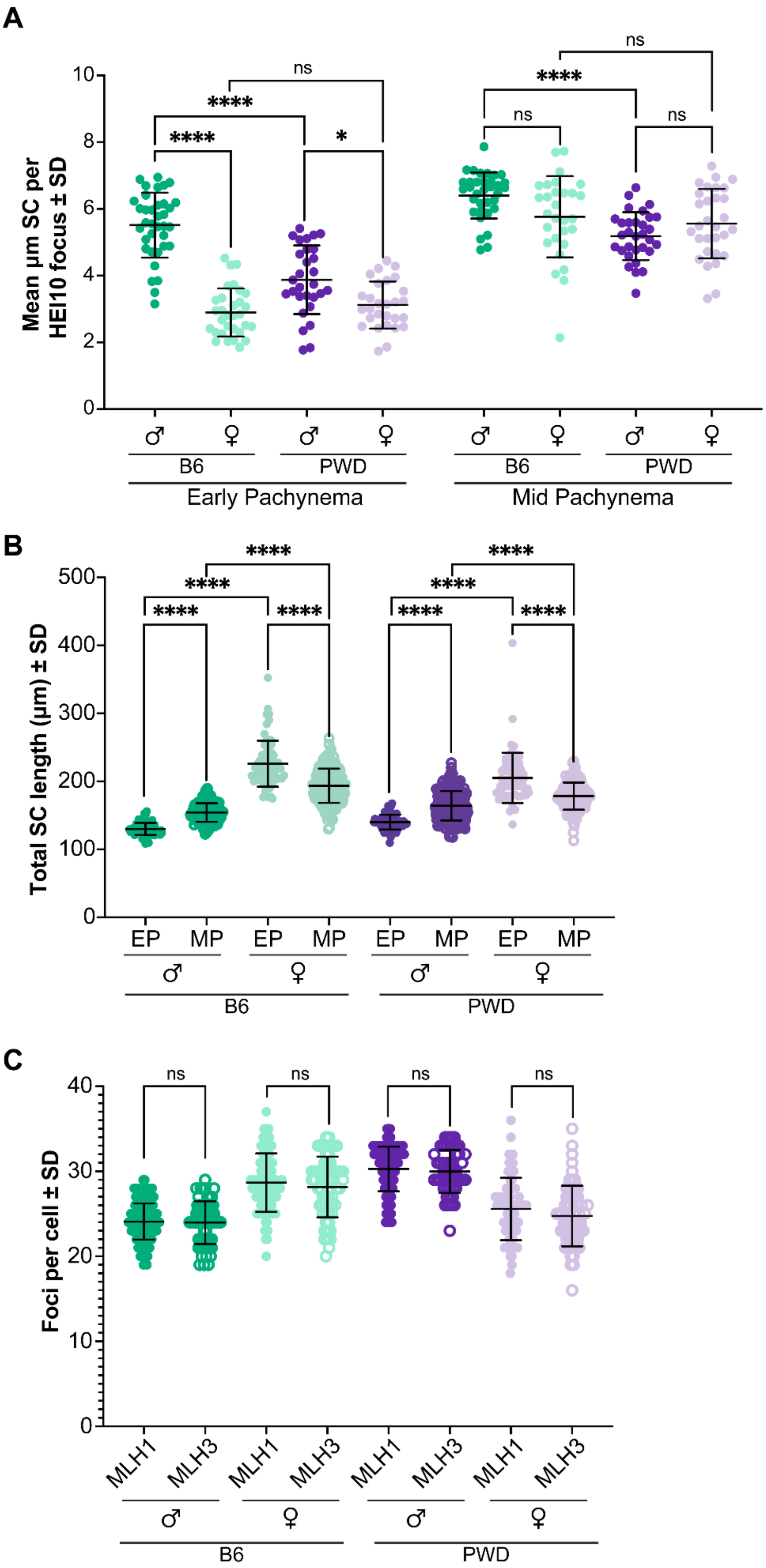
A
Mean µm SC per HEI10 focus ± SD
ns
****
****
*
ns
****
ns
ns
♂
♀
B6
PWD
Early Pachynema
Mid Pachynema
B
Total SC length (µm) ± SD
****
EP
MP
C
Foci per cell ± SD
ns
MLH1
MLH3

Fig. S6 Sex differences in HEI10 foci density, SC lengths differ between early and mid-pachynema, and MLH1 vs. MLH3 foci in mid-pachynema. (A) Mean microns of SC per HEI10 focus for B6 and PWD males and females at early and mid-pachynema. Significant sex differences in mean microns/focus were observed only at early pachynema in B6 (early: $t = 12.6$; mid: $t = 2.5$) and PWD (early: $t = 3.25$; mid: $t = 1.64$). Significant strain differences were only observed between males (early: $t = 6.54$; mid: $t = 7.01$); samples sizes (animals [cells]): For early pachynema: B6 male (3 [36]), female (3 [31]); PWD male (3 [29]), female (4 [28]); For mid pachynema: B6 male (3 [35]), female (3 [29]); PWD male (3 [31]), female (3 [30]). (B) Total SC length at pachynema ($F = 165.3$, $p<0.0001$) is shorter at early (closed circles) versus mid (open circles) stages for B6 ($t = 17.98$) and PWD ($t = 12.64$) males, but longer at early pachynema for B6 ($t = 7.12$) and PWD ($t = 5.45$) females. Total SC length is consistently greater in females than males for B6 (early: $t = 22.66$; mid: $t = 18.24$) and PWD (early: $t = 13.62$; mid: $t = 6.4$); samples sizes (animals [cells]): For early pachynema: B6 male (4 [79]), female (4 [67]); PWD male (4 [72]), female (8 [64]); For mid pachynema: B6 male (8 [229]), female (9 [164]); PWD male (8 [233]), female (5 [136]). (C) For B6 and PWD male and female mid-pachytene cells, mean MLH1 foci number (closed circles) does not differ significantly from mean MLH3 foci number (open circles); samples sizes (animals [cells]): For MLH1 B6 male (7 [200]), female (4 [104]); PWD male (7 [203]), female (3 [74]); For MLH3 B6 male (5 [64]), female (6 [60]); PWD male (5 [51]), female (4 [62]). Asterisks denote significant differences: **** $p < 0.0001$, * $p < 0.05$ (Games-Howell post-hoc). Error bars represent SD.

Supplemental Table Legends

Table S1 Full one-way ANOVA analyses

Summary of Brown-Forsythe ANOVA test and subsequent Games-Howell's multiple comparison test for significant differences in mean SC length, MLH1 foci number, microns SC per MLH1 focus, and chiasmata number between all sexes and strains.

Table S2 Multiple linear regression analysis of the effects of sex and total SC length on per-nucleus class I CO rates

Adj. R^2 denotes the proportion of variance in MLH1 foci number explained by SC length and sex (females as reference). Regression coefficients represent the change in MLH1 foci per one-unit change in either sex (male) or SC length (micron) holding other variables constant. Standardized regression coefficients (β) indicate the magnitude of each predictor's effect (SC length or sex) on MLH1 foci number in common units (standard deviation).

Table S3 Logistic regression predicting the likelihood of an SC having multiple MLH1 foci

B6 female mice were used as a reference group. Odds ratios greater than 1 indicate increased likelihood of an SC having more than one MLH1 focus with a change in the respective variable (increasing SC length or change in strain or sex) while holding all over variables constant. Model fits were evaluated using G^2 (likelihood ratio test statistic) and several pseudo-R^2 values. Both the main effects only model and the full model (with all interaction terms) showed significantly better fits than the null model (no predictors), and the full model showed marginally better fit than the main effects only model.

Table S4 Full analysis of distance between adjacent MLH1 foci

Both the absolute (microns) and normalized (%SC length) distance between adjacent MLH1 foci on the same SC were analyzed by Kolmogorov-Smirnov (KS) test using pooled data from all

SCs, the 5 longest SCs per cell, and the 5 shortest SCs per cell. Top table summarizes median inter-focus distance, and the bottom table summarizes the KS test statistics and their respective Bonferroni-adjusted p-values.

Table S5 Full analysis of distance from the centromere to the first MLH1 focus

Both the absolute (microns) and normalized (%SC length) distance between the centromere and the first MLH1 focus for each SC were analyzed by Kolmogorov-Smirnov (KS) test using pooled data from all SCs. The analysis was also performed for SCs with 1 MLH1 focus and multiple MLH1 foci. Top table summarizes the median focus distance from the centromere, and the bottom table summarizes the KS test statistics and their respective Bonferroni-adjusted p-values.

Table S6 List of antibodies used in immunofluorescence staining experiments
